# Quercetin as a Promising Antiprotozoan Phytochemical: Current Knowledge and Future Research Avenues

**DOI:** 10.1155/2024/7632408

**Published:** 2024-02-29

**Authors:** Hamed Memariani, Mojtaba Memariani, Abdolmajid Ghasemian

**Affiliations:** ^1^Department of Medical Microbiology, Tehran University of Medical Sciences, Tehran, Iran; ^2^Noncommunicable Diseases Research Center, Fasa University of Medical Sciences, Fasa, Iran

## Abstract

Despite tremendous advances in the prevention and treatment of infectious diseases, only few antiparasitic drugs have been developed to date. Protozoan infections such as malaria, leishmaniasis, and trypanosomiasis continue to exact an enormous toll on public health worldwide, underscoring the need to discover novel antiprotozoan drugs. Recently, there has been an explosion of research into the antiprotozoan properties of quercetin, one of the most abundant flavonoids in the human diet. In this review, we tried to consolidate the current knowledge on the antiprotozoal effects of quercetin and to provide the most fruitful avenues for future research. Quercetin exerts potent antiprotozoan activity against a broad spectrum of pathogens such as *Leishmania* spp., *Trypanosoma* spp., *Plasmodium* spp., *Cryptosporidium* spp., *Trichomonas* spp., and *Toxoplasma gondii*. In addition to its immunomodulatory roles, quercetin disrupts mitochondrial function, induces apoptotic/necrotic cell death, impairs iron uptake, inhibits multiple enzymes involved in fatty acid synthesis and the glycolytic pathways, suppresses the activity of DNA topoisomerases, and downregulates the expression of various heat shock proteins in these pathogens. In vivo studies also show that quercetin is effective in reducing parasitic loads, histopathological damage, and mortality in animals. Future research should focus on designing effective drug delivery systems to increase the oral bioavailability of quercetin. Incorporating quercetin into various nanocarrier systems would be a promising approach to manage localized cutaneous infections. Nevertheless, clinical trials are needed to validate the efficacy of quercetin in treating various protozoan infections.

## 1. Introduction

Protozoan infections continue to exact a heavy toll on public health in underdeveloped countries. In 2021, the number of malaria cases was estimated to be 247 million, resulting in 619,000 deaths. Approximately 700,000 to 1 million new cases of leishmaniasis occur per year, and 1 billion people who live in endemic areas are at risk of infection [1]. The global distribution of countries reporting cases of malaria or leishmaniasis demonstrates an overlap of these diseases in Central and South America, Africa, and South Asia [1]. In the African region, about half of the malaria cases globally were found in countries such as Nigeria, the Democratic Republic of Congo, Uganda, Angola, Burkina Faso, and Mozambique [2]. In 2022, about 85% of global visceral leishmaniasis (VL) cases were reported from seven countries: Brazil, Ethiopia, India, Kenya, Somalia, South Sudan, and Sudan. The highest reported cases of cutaneous leishmaniasis (CL) worldwide is observed in Afghanistan, Algeria, Brazil, Colombia, Iran, Iraq, Peru, and the Syrian Arab Republic [3]. Due to underreporting, however, there is no reliable way to estimate the true burden of all protozoan infections.

Despite tremendous progress in the prevention and treatment of infectious diseases over the past century, only few antiparasitic drugs have been developed so far. Agents for satisfactory treatment of certain parasitic infections, particularly African trypanosomiasis (sleeping sickness) and Chagas' disease, are still lacking [4]. The problem is further aggravated by the intrinsic toxicity of the antiparasitic drugs. Another issue concerning these medications is the development of drug resistance in protozoan pathogens (e.g., chloroquine in *Plasmodium*, metronidazole in anaerobic parasites, sulfonamide in *Toxoplasma gondii*, and diloxanide for intestinal protozoa) [5]. Therefore, there is an urgent need to find novel chemotherapeutic agents with low toxicity while maintaining high antiparasitic efficacy. Since natural products from animal and plant sources represent an inexhaustible repertoire of antimicrobials, they have long enticed a great interest among researchers [6, 7].

The therapeutic properties of plants have been recognized since time immemorial, and even today, they remain an essential source for identifying new potential drugs [8]. Salient examples of plant-derived natural compounds that have become indispensable for modern pharmacotherapy can be found in the field of anticancer drugs such as paclitaxel, vincristine, vinblastine, and camptothecin. The historical relevance of plant-derived compounds in the development of antimalarial medications including quinine and artemisinin implies that botanicals represent an important source of new antimicrobial agents [8].

Plant secondary metabolites are key compounds that bestow upon plants their color, flavor, and odor, as well as mediating plant responses to unfavorable environmental conditions [9]. Polyphenols are secondary metabolites that are found ubiquitously across plants. These compounds are involved in pigmentation, reproduction, and protection against phytopathogens. Flavonoids are the most abundant polyphenols with robust antioxidant properties that are ingested in large quantities as part of the human diet [10].

Quercetin (3,3′,4′,5,7-pentahydroxyflavone; Que) is one of the most commonly consumed flavonoids that can be found in a variety of edible vegetables and fruits. Que has a bitter taste and is poorly soluble in water, but is quite soluble in alcohol and lipids. Its poor aqueous solubility, chemical instability, and short biological half-life may decrease its efficacy in food and pharmaceuticals [11]. Que usually exists as sugar conjugates such as rutin, quercitrin, or isoquercetin. Its bioavailability is influenced by the type of glycosides found in different food sources. Only the free form of Que was believed to be absorbed at the intestinal level through passive diffusion because of its hydrophobic nature, but later studies revealed that the adsorption of Que glycosides nearly doubles that of its corresponding aglycon [12].

Que has a wide range of biological properties, including anticancer, anti-inflammatory, antidiabetic, antibacterial, antiviral, cardioprotective, neuroprotective, antiaging, and rejuvenating properties, making it potentially useful for drug development [13, 14]. To our knowledge, no attempt has been made to review antiprotozoan properties of Que. Thus, this review is the first to summarize the current state of knowledge regarding both in vitro and in vivo effects of Que on protozoan pathogens of medical and veterinary importance, with an emphasis upon underlying mechanisms of action. Major features of the relevant pathogens are also briefly described.

## 2. *Leishmania* spp.

Leishmaniasis is a neglected vector-borne disease, caused by obligate intracellular kinetoplastids of the genus *Leishmania* [15]. The pathogen has a dimorphic life cycle that alternates between an invertebrate vector and a mammalian host. Extracellular flagellated promastigotes exist in the alimentary tract of sandflies, whereas nonmotile, round-shaped amastigotes multiply within host cells [16]. The clinical forms of leishmaniasis are largely dependent on the parasite species that show tropism for either skin or viscera, as well as the genetic background, nutritional status, and immunocompetence of the host. The broad spectrum of clinical manifestations ranges from self-healing cutaneous lesions to disfiguring mucocutaneous lesions and even to life-threatening systemic infections [17]. For instance, *Leishmania tropica*, *Leishmania major*, and *Leishmania aethiopica* are the main causative agents of the Old World CL, while *Leishmania donovani* and *Leishmania infantum* (both in the Old World) or *Leishmania chagasi* (in the New World) can cause a serious visceral disease (commonly known as kala-azar). In the New World, *Leishmania braziliensis* is responsible for severe forms of cutaneous and mucocutaneous infections [15].

Treatment of human leishmaniasis is a challenging issue, and no vaccine has yet been approved for commercial use against any form of the disease [18]. Pentavalent antimonials such as sodium stibogluconate (SSG, Pentostam®) and meglumine antimoniate (Glucantime®) have been the mainstay of therapy for decades. These agents seem to inhibit bioenergetic pathways, especially glycolysis and fatty acid oxidation, in *Leishmania* amastigotes. Growing rates of drug resistance to antimonials in conjunction with their intrinsic toxicity have necessitated the development of new drugs with novel targets [5].

### 2.1. Effects of Que on *Leishmania amazonensis*

Several studies demonstrated that Que treatment caused a dose-dependent reduction in the viability of *L. amazonensis* in vitro. Notably, Montrieux et al. [19] found that Que was more potent than the reference drug Glucantime® in inhibiting the growth of *L. amazonensis* promastigotes and amastigotes. The available evidence [19, 20] also indicates that Que has a higher specificity for intracellular amastigotes than peritoneal macrophages (Tables 1 and 2).

The study by Fonseca-Silva et al. [20] provided mechanistic insight into how Que kills *L. amazonensis*. In this respect, reactive oxygen species (ROS) production and dysfunction of mitochondrial membrane potential (ΔΨ_*m*_) could play a pivotal role in Que-evoked cell death in *L. amazonensis*. A separate study also found that Que is a mixed inhibitor of *L. amazonensis* arginase (ARG-L). This metalloenzyme catalyzes the hydrolysis of L-arginine to urea and L-ornithine, providing a substrate for polyamine biosynthesis [23]. Polyamines are indeed necessary for the cell proliferation and the production of trypanothione, a low-molecular-mass dithiol that is used by specific enzymes to neutralize the ROS generated by the macrophages during infection [69]. According to structure–activity relationship analyses of several dietary flavonoids, such as Que, the hydroxyl group at position 3 is involved in arginase inhibition, whereas the hydroxyl group at position 5 is not. The presence of the catechol group appears to be a key feature of ARG-L inhibitors [70]. Docking simulations also demonstrated that the catechol group of Que interacts with Asp-29, contributing to the formation of a metal bridge for the cofactors Mn^2+^_A_ and Mn^2+^_B_ in the active site of ARG-L [23]. Since ARG-L is the first enzyme in the polyamine pathway, its inhibition by Que could cause oxidative stress due to insufficient production of trypanothione [71].

Studies in BALB/c mice revealed that daily oral doses of Que (16 mg/kg body weight) for 30 and 51 days reduced the lesion sizes and parasite loads [72, 73]. Surprisingly, oral Que was superior to intraperitoneal SSG in decreasing parasite loads (76% vs. 62%) [72]. Since the parasite burden was examined 30 days after drug withdrawal, Que appeared to have a long-lasting activity, at least in CL [72]. Following five intralesional injections of Que (30 mg/kg) every 4 days, a significantly lower parasite burden (*p* < 0.05) was observed at 4 and 6 weeks postinfection in comparison to the vehicle-treated or untreated groups [19].

When encapsulated in lipid-core nanocapsules (LNCs) of poly-*ε*-caprolactone, 0.4 mg/kg of oral Que was sufficient to significantly (*p* < 0.01) decrease lesion sizes as compared to free (noncapsulated) Que [73]. This result indicates a notable increase in the potency of Que after encapsulation. It seems that the LNC polymeric shell protects Que against gastric and intestinal degradation, allows for a better drug absorption, and may consolidate structural integrity in blood circulation. There were no compound-related clinical signs of toxicity (Table 3). Based on these observations, nanoencapsulation in LNC could be considered as a new and safe strategy to improve the oral efficacy of Que against CL.

### 2.2. Effects of Que on *Leishmania braziliensis*

A recent study demonstrated that Que had a dose-dependent cytotoxicity against both *L. braziliensis* promastigotes and amastigotes [28]. However, several studies have shown that Que had lower antileishmanial activity than amphotericin B [24, 27], SSG [25], and miltefosine [28]. Compared to mammalian cell lines, *Leishmania* promastigotes are more susceptible to the inhibitory effects of Que (Table 2).

The couple tryparedoxin/tryparedoxin peroxidase uses trypanothione as a source of electrons to neutralize the hydrogen peroxide produced by the macrophages during infection. This detoxification pathway is an attractive drug target because it is indispensable for parasite survival and absent in the human host [81]. Using in silico docking analysis, the binding energy score between modeled *L. braziliensis* tryparedoxin peroxidase (Try P) and Que was calculated as −11.8601 kJ/mol. Accordingly, Que seems to have strong binding interactions with *L. braziliensis* Try P. A further finding was that the amino acids Pro-11, Asp-134, and Lys-136 in Try P were shown to interact with Que [82]. These data provided initial insights into the potential of Que as a Try P inhibitor.

As reported by Cataneo et al. [26], Que promoted promastigote killing through upregulation of ROS, phosphatidylserine externalization, and loss of plasma membrane integrity, which are evocative of dual modes of apoptotic/necrotic death in Que-treated promastigotes. Que was also capable of modulating cytokines, decreasing tumor necrosis factor-alpha (TNF-*α*), and increasing interleukin 10 (IL-10) production without changing nitric oxide (NO) levels [26]. It was proposed that the mechanisms contributing to *L. braziliensis* eradication by Que were independent of the oxidative burst activation. It is worth mentioning that NO generation by the inducible nitric oxide synthase (iNOS) plays a key part in controlling infections caused by *Leishmania* parasites. Indeed, various stimuli such as interferon-gamma (IFN-*γ*), IL-1*β*, TNF-*α*, and parasitic/bacterial infections induce iNOS expression in macrophages [83].

Iron starvation could be considered as an ideal therapeutic strategy to control leishmaniasis. Que was shown to reduce the labile iron pool, increase iron bound to transferrin, and upregulate both nuclear factor erythroid 2-related factor 2 (Nrf2) and heme oxygenase-1 (HO-1) expressions [26]. The transcription factor Nrf2 plays a central role in augmenting antioxidative defense and functions as a modulator of iron signaling by regulating the expression of various genes such as HO-1, ferroportin, and ferritin [84]. In congruence with these results, one study showed that free Que can gain access to the cytosol where it shuttles labile iron from cell compartments followed by its transfer to transferrin [85]. Taken together, Que appears to impair iron uptake by the parasite, besides acting as an iron chelator.

In a recent study, treatment of *L. braziliensis*-infected hamsters with oral Que (20 mg/kg, five times a week), for eight weeks starting from the first week of infection, significantly decreased lesion thickness and parasite load [28]. However, oral Que exhibited lower in vivo efficacy compared to intraperitoneal Glucantime® (80 mg/kg, three times a week). Treatment of the hamsters with Que for eight weeks did not alter the levels of creatinine, alanine transaminase, and aspartate transaminase when compared to the untreated animals, indicating that Que had no renal and hepatic toxicity. Further research is needed to confirm and extend these results.

### 2.3. Effects of Que on *Leishmania donovani*

Que was shown to exert a dose-dependent inhibitory effect on the growth of both *L. donovani* promastigotes and amastigotes in vitro [29]. Que has superior selectivity for *L. donovani* amastigotes over the mammalian cells (Tables 1 and 2). In comparison to standard drugs (miltefosine, pentamidine, and SSG), Que showed weaker growth inhibitory activities toward *L. donovani* [30, 32, 35].

Que is able to arrest cell-cycle progression, leading to increased apoptotic cell death [29, 35]. Que can also induce DNA damage and NO production [35], both of which are thought to play a regulatory role in apoptosis in *Leishmania* [86]. Nuclear condensation, appearance of lipid reservoirs, and disruption of the mitochondrion–kinetoplast complex are other effects of Que on *L. donovani* promastigotes. The appearance of lipid reservoirs could probably result from the entry of substance into vacuoles by simple diffusion and/or production of abnormal lipids in response to Que treatment [35]. The latter might distort the flagellar pockets. Besides inducing morphological changes, the expression levels of both trypanothione reductase and trypanothione synthetase were found to be downregulated in the Que-treated parasites. It should not be forgotten that both of these two enzymes are unique to *Leishmania* and are crucial for the parasite survival [43]. In summary, Que appears to work simultaneously on various targets in *Leishmania*, culminating in cell death.

Que is capable of intercalating into the DNA [31], inducing double-stranded DNA damage [35], and inhibiting both catalytic activity of topoisomerase II and DNA synthesis in vitro [29]. In one study, exposure of *L. donovani* promastigotes to Que caused a drastic increment in total mass of kinetoplast DNA (kDNA) minicircles containing nicks/gaps and linearized minicircle molecules, which were generated by topoisomerase II-mediated double-strand cleavage of minicircles from the kDNA network [29]. In another study [31], Que was found to be a potent inhibitor of the recombinant *L. donovani* topoisomerase I. This effect appears to arise from stabilization of the topoisomerase I–DNA cleavage complexes, which impedes the subsequent religation step. In light of these findings, topoisomerase inhibition seems to be one of the major mechanisms responsible for antileishmanial activity of Que.

Iron and heme are necessary for various conserved metabolic pathways such as electron transport and signal transduction. Since *Leishmania* lacks cytosolic iron storage proteins and is a heme auxotroph, the parasite must acquire nutritional iron and heme from its host [87]. Given the importance of iron acquisition for survival and pathogenicity of *Leishmania* parasites, iron deprivation might be considered as an effective strategy to control leishmanial infections. Evaluation of the interaction between Fe^3+^ and Que revealed a metal-chelating ability of Que [76]. The observed Fe^3+^-reducing ability of the flavonoids could be ascribed to the catechol structure of the B ring and the presence of a 3-hydroxy group in the C ring [88]. Moreover, Que appears to limit the availability of Fe to intraphagosomal parasites by decreasing Fe distribution in peritoneal macrophages [76]. Thus, Que could interfere with iron metabolism in *L. donovani*.

Recently, the combination of antiparasitic drugs with nanocarriers has become a promising strategy for the treatment of leishmaniasis [89]. In this regard, Que-conjugated gold nanoparticles (QAunp) have proved to be highly effective against both axenic and intracellular *L. donovani* amastigotes [34]. In particular, QAunp was superior to Que alone in inhibiting the growth of wild type, SSG-resistant, and paromomycin-resistant strains of *L. donovani* (Table 1). It seems that gold nanoparticles impair the parasite's oxygen metabolism. When combined with Que, gold nanoparticles act synergistically to potentiate the activity of Que against the pathogens in macrophages. Another finding worthy of mention is the trivial toxicity of both Que and QAunp against murine peritoneal macrophages, underpinning the safety of these agents (Table 2). In fact, the use of nanocarriers offers a number of advantages, such as reduction of drug toxicity, enhancement of treatment efficacy, improvement of selectivity, modulation of the drug pharmacokinetics, drug solubilization enhancement, protection of drugs against degradation, and sustained drug release directly at the site of action [89].

In golden hamsters infected with amastigotes of *L. donovani*, orally administered Que (14 mg/kg body weight) was remarkably efficient in reducing the splenic parasite burden [29]. When administered intraperitoneally to BALB/c mice, Que (30 mg/kg of body weight/day) was demonstrated to diminish the hepatic burden of *L. donovani* [32]. Consistent with these findings, others [74, 76] reported high potency of Que in reducing parasitemia in the spleens of the infected animals. In one study, Sarkar et al. [77] intercalated Que into different vesicular suspensions (Table 3), with the aim of boosting its efficacy and reducing its in vivo toxicity. With an 87% reduction in the splenic parasite burden, Que-intercalated nanoparticles were the most effective treatment, followed by Que-intercalated niosomes (68%). In comparison, a reduction in splenic burden by approximately one-quarter was evident in animals exposed to free Que. It was realized that smaller vesicles could be more effective than the larger ones. Nanocapsulated Que was the most effective in mitigating both hepatotoxicity and renal toxicity as compared with other tested vesicular forms and free Que [77]. Based on these data, it remains to be determined whether such formulations are clinically advantageous in the treatment of leishmaniasis.

Sen et al. [76] reported that the combination treatment with Que and serum albumin (Salb) led to a decreased incorporation of ^59^Fe in the amastigotes collected from infected hamsters. This combination remarkably reduced the activity of ribonucleotide reductase (RR) in *L. donovani* amastigotes isolated from infected hamsters [76]. The reduction in the activity of RR seems to be associated with the Que-mediated decrease in Fe acquisition by the amastigotes. RR is an iron-containing enzyme that catalyzes the rate-limiting step in the de novo synthesis of DNA building blocks, thereby playing a key role in cellular proliferation [90]. Overall, Que may be able to prevent leishmanial growth by interfering with iron metabolism and targeting RR.

Considering the antioxidant properties of flavonoids, Sen et al. [74] investigated the ability of five flavonoids (i.e., Que; rutin; hesperidin; 5-hydroxy 3,6,7,3′,4′-pentamethoxy flavone; and diosmin) to control VL-associated anemia in golden hamsters. Que was the most effective of all agents tested in dampening the oxidation of both lipids and proteins on the membranes of red blood cells (RBCs) in *L. donovani*-infected hamsters [74]. It is noteworthy that lipid peroxidation causes the production and dissemination of lipid radicals, oxygen uptake, rearrangement of the double bonds in unsaturated lipids, and eventual damage to the RBC membrane lipids [91]. Que also excelled as the most potent flavonoid compared to others in rectifying VL-associated anemia in hamsters. In this connection, decrements in both hemoglobin (Hb) level and RBC half-life due to VL were remarkably reversed by Que treatment [74]. The greater number of hydroxyl groups and the presence of 3-hydroxyl in Que could explain the superiority of Que over the studied flavonoids in decreasing both oxidative hazards and premature destruction of RBCs.

Combination therapy for VL has been advocated as an auspicious approach to improve treatment efficacy and tolerability, to reduce treatment duration and expenditure, and to prevent the emergence of drug resistance [92]. In one study [74], treatment of *L. donovani*-infected hamsters with a combination of SSG and Que was more successful in decreasing hydroxyl radical production (57.9%) in RBCs than either Que (47.4%) or SSG (23.7%) alone. Examining the protein profile of the RBC membrane in the infected animals also revealed a better efficacy of this combination in preventing proteolytic degradation compared to single therapy. Additionally, simultaneous treatment of the infected animals with both agents demonstrated greater effectiveness in replenishing decreased Hb levels, reversing shortened RBC lifespan, and restoring the Salb deficit caused by *L. donovani* [74]. VL appears to be associated with serum hypoalbuminemia, which in turn may weaken the potency of Que against the disease [74]. With this in mind, Sen et al. [75] sought to answer the question of whether a Que/hamster Salb combination could aggrandize the in vivo bioavailability of Que. Compared with Que alone, the combination therapy caused a gradual increase in Que levels in the cytosol of RBCs collected from *L. donovani*-infected animals. Likewise, in another study, an increased bioavailability of Que content in the liver of infected animals was also achieved with the combination treatment [76]. The combination of Que with Salb killed *L. donovani* more potently than Que alone, as judged by lower splenic parasite loads in animals receiving the combination treatment [76]. Concurrent use of Que and hamster Salb was also found to be more effective than Que alone in reducing cellular iron decompartmentalization and hydroxyl radical production in RBCs, thereby enhancing the lifespan of hamster RBCs during infection [75]. Cumulatively, the combination of Que with other drugs/carriers may well be viewed as a protective measure against premature hemolysis by free radicals during VL.

### 2.4. Effects of Que on *Leishmania infantum*

Lately, Garcia et al. [37] have shown that several natural phenolic substances, such as Que, were able to impede the in vitro activity of *L. infantum* arginase (ARGLi). Investigation of the structural characteristics of the phenolic compounds with potent inhibitory activity on ARGLi revealed that they all possess a catechol group [37]. Clearly, further research should be undertaken to advance our knowledge about the structure-activity relationship of the phenolic substances.

Encapsulation of Que into poly-*ε*-caprolactone (PCL) nanoparticles seems to be an effective and safe approach to enhance the antileishmanial activity of Que in vitro [36]. Compared to Que alone, Que-loaded PCL nanoparticles (QPNPs) exhibited not only higher killing activities against *L. infantum* promastigotes and intracellular amastigotes (Table 1) but also lower cytotoxicity toward murine macrophages (Table 2). It would therefore be desirable to determine whether Que-loaded nanoparticle formulations could be effective against *Leishmania* infections in animal models.

### 2.5. Effects of Que on *Leishmania major*

Recent in vitro studies have shown that Que eliminates *L. major* promastigotes in a dose- and time-dependent manner (see Table 1). When *L. majo*r promastigotes were exposed to Que (400 *μ*M) for 24 h, they underwent morphological changes reminiscent of necrosis and, to a lesser extent, apoptosis. Upon further experimentation, it was found that Que instigates a protease-independent programmed cell death in the parasites [10].

Another important in vitro finding is that Que has greater activity against *L. major* than Glucantime® [38, 41]. In another study, Que-capped silver nanoparticles exhibited substantially higher leishmanicidal activities against *L. major* promastigotes than Que alone or Glucantime® [42]. Undoubtedly, nanocarriers hold great potential for improving the antileishmanial activity of Que.

In an in vivo study, Hamidizadeh et al. [78] reported a higher percentage of recovered animals receiving Que (14 mg/kg) through different routes of administration compared with the Glucantime®-treated group. Nevertheless, this difference was not statistically significant, which could be due to the small number of mice tested. Using the murine air pouch mode that mimics the phlebotomine infection in BALB/c mice, Que was shown to reduce neutrophil influx in the air pouch cavity at 24 h postinfection. However, the density of resident macrophages in Que-treated infected mice was not statistically different from that in the untreated infected animals [10]. It is worth mentioning that the recruitment of a specific cell population after *Leishmania* infection can affect the outcome of the disease [93].

Reductions in both lesion size and inflammatory responses, along with acceleration of wound healing, were observed in Que-treated animals infected with *L. major* [40–42, 94]. In one study, the abundance of apoptotic neutrophils containing apoptotic amastigotes in Que-treated mice was noticed [10], suggesting that exposure to Que could markedly abrogate *L. major*-induced apoptosis delay. Furthermore, Que restored ROS generation and TNF-*α*-induced iNOS activity in subcutaneous tissues of BALB/c mice at 24 and 96 h after *L. major* infection [10]. Overall, Que may hold promise for the treatment of uncomplicated CL.

### 2.6. Effects of Que on *Leishmania tropica*

A recent study found that *L. tropica* amastigotes and promastigotes, unlike human RBCs, were susceptible to Que [43]. Que-treated promastigotes also exhibited clear signs of DNA fragmentation, one of the biochemical hallmarks of apoptosis [86]. It is possible that DNA damage-associated apoptosis could be the cause of the observed loss of parasite viability.

In silico docking analysis unveiled that Que could be seated appropriately inside the binding pocket of both trypanothione reductase and trypanothione synthetase [43]. Further in silico evidence in favor of this finding was obtained by molecular dynamics simulations, indicating strong interaction between Que and both enzymes [43]. It should be noted that both trypanothione reductase and trypanothione synthetase play a pivotal role in maintaining leishmanial growth and do not exist in human cells.

## 3. *Trypanosoma* spp.

Like *Leishmania*, *Trypanosoma* is a kinetoplastid protozoan. Trypanosomiasis is one of the neglected tropical diseases [95]. Poor and marginalized populations are the primary victims of the diseases. *T. cruzi* is responsible for Chagas' disease (American trypanosomiasis) in Latin America, whereas *T. brucei* causes human African trypanosomiasis (HAT) or sleeping sickness in East and West Africa. These parasites have digenetic life cycles that involve an invertebrate vector (a triatomine bug infected with *T. cruzi* or a tsetse fly infected with *T. brucei*) and a mammalian host. For both diseases, treatment is available, but sometimes, a cure cannot be achieved [96].

Nifurtimox and benznidazole are the only approved parasiticidal drugs for the treatment of Chagas' disease. They are highly effective in treating acute and recent infections, as well as in preventing maternal-fetal transmission, but their effectiveness declines with chronic infection. Both drugs are also fraught with adverse clinical effects. As for *T. brucei*, only four drugs are registered for the treatment of early- and late-stage HAT: pentamidine, suramin, melarsoprol, and eflornithine [5].

### 3.1. Effects of Que on *Trypanosoma brucei*

In vitro evidence suggests that Que is far more toxic to bloodstream trypomastigote forms of *T. b. brucei* than different mammalian cells (Tables 1 and 2). Que also appears to induce dose- and time-dependent apoptosis in *T. b. gambiense*. In contrast to *T. b. gambiense*, no clear evidence of apoptosis in Que-treated human normal leukocytes was reported, whether activated by parasite-soluble factors or not [46].

Trypanosome infection instigates the rapid production of inflammatory components such as TNF-*α* and ROS/RNS like NO. Classically activated macrophages are known as the major effector cells against trypanosomes, relying on ROS/RNS production, trypanolytic function of soluble TNF, and parasite engulfment [97]. Mamani-Matsuda et al. [46] found that Que markedly hampered TNF-*α* production in human macrophages that had already been activated by either anti-CD23 monoclonal antibody (mAb) or *T. b. gambiense*. In the presence of Que, a substantial decrease in NO production was also observed in anti-CD23 mAb-activated macrophages [46]. Although macrophage-derived TNF-*α* and NO are involved in trypanocidal activity, chronic overexpression of these mediators may contribute to the pathophysiology of HAT. Thus, Que seems to be helpful in the amelioration of inflammation during trypanosome infection. However, the mechanisms underlying the anti-inflammatory actions of Que in macrophages still remain a mystery and deserve further investigation. Overall, Que could be of potential use in the treatment of HAT owing to its anti-inflammatory and trypanocidal effects.

Kinetoplastids require keeping their proteome function in response to different stress factors. To this end, heat shock proteins (Hsps), whose main function is to facilitate proteostasis, play a crucial role in the survival and cell stage differentiation. Several Hsp chaperones and cochaperones have been characterized in kinetoplastids and classified based on their molecular masses, such as Hsp110, Hsp90, Hsp70, Hsp60, and Hsp40 [98]. Proteins belonging to the Hsp70 class aid in coordinating multiple key cellular processes, including the folding and assembly of newly synthesized proteins, the refolding of misfolded and aggregated proteins, and the proteolytic degradation of denatured or unstable proteins [99]. Genome annotation revealed that *T. brucei* harbors a dozen Hsp70 chaperones [100]. TbHsp70.c is a Hsp70 from *T. brucei*, whose expression levels were previously shown to be upregulated in response to heat stress. TbHsp70.c also acts as a holdase, suppressing protein aggregation. Cytosol-localized Tbj2 was shown to increase the ATPase activity of TbHsp70.c, suggesting that it may function as a cochaperone of TbHsp70.c [101]. Que also inhibited the ATPase activity of TbHsp70.c, in either the presence or absence of Tbj2. Molecular docking analysis suggests that Que can bind to the nucleotide binding pocket of TbHsp70.c [101]. Notwithstanding this, further research is necessary to corroborate these findings.

*T. brucei* exclusively uses glycolysis to generate ATP in the mammalian bloodstream [102]. Hexokinase catalyzes the first step in glycolysis, facilitating the transfer of the *γ*-phosphoryl group of ATP to glucose for producing glucose-6-phosphate [103]. The *T. brucei* genome encodes two hexokinases, namely, TbHK1 and TbHK2, that are 98% identical in terms of amino acid sequence. These enzymes have been detected in the glycosomes of both bloodstream and procyclic forms of the parasites [103]. Larit et al. [39] utilized molecular docking to show that there is a strong affinity between Que and TbHK1. They proposed that TbHK1 could be a potential target of Que. Intriguingly, Que could act as a mixed inhibitor of recombinant TbHK1 with respect to ATP [44]. Spectroscopic analysis showed that Que quenches the emission of Trp-177, which is located close to the hinge region of this enzyme. Que also appeared to partially accumulate in glycosomes, the subcellular home of TbHK1. Manipulated procyclic *T. brucei* cells overexpressing TbHK1 were more resistant to the inhibitory effects of Que as compared with the Que-treated control parasites, whereas RNA interference-mediated silencing of TbHK1 expression in *T. brucei* cells rendered them more sensitive to the compound [44]. Indeed, further experiments are needed to better understand how changes in the expression levels of TbHK1 influence the vulnerability of *T. brucei* cells to Que.

### 3.2. Effects of Que on *Trypanosoma cruzi*

Two studies [21, 48] demonstrated that Que has antiprotozoal activity against *T. cruzi* trypomastigotes (see Table 1). Nevertheless, the inhibitory activity of Que was weaker than that of benznidazole [32]. Research is needed to determine whether Que works synergistically in combination with antitrypanosomatid drugs.

Oxidative phosphorylation in *T. cruzi* is mediated by a mitochondrial Mg^2+^-stimulated adenosine triphosphatase (ATPase), similar to all other known eukaryotic or prokaryotic systems [104]. In vitro evidence suggests that Que acts as an inhibitor of soluble and membrane-bound mitochondrial ATPases from *T. cruzi* [47]. Therefore, Que has the potential to disrupt mitochondrial energy metabolism by inhibiting *T. cruzi* mitochondrial ATPase. A deeper understanding of mitochondrial enzymes in trypanosomes would clearly advance our efforts to develop new antitrypanosomatid drugs.

As previously mentioned, bloodstream trypomastigotes are highly dependent on glycolysis for energy generation because they lack a functional Krebs cycle and mitochondrial respiratory chain [105]. This dependence on glycolysis as a source of energy marks glycolytic enzymes of *T. cruzi* as potential new drug targets. Glyceraldehyde-3-phosphate dehydrogenase (GAPDH) is one of the major enzymes in the glycolytic pathway that catalyzes the reversible oxidative phosphorylation of D-glyceraldehyde-3-phosphate to 1,3-*bis*-phospho-D-glyceric acid in the presence of NAD^+^ and inorganic phosphate. Inhibition of the glycosomal GAPDH would preclude *T. cruzi* from being infective [106]. It is worth mentioning that a substantial decrease in ATP supply due to specific inhibition of GAPDH would result in the rapid death of *T. cruzi* [107]. Freitas and co-workers [49] also proposed Que as an inhibitor of *T. cruzi* GAPDH. These findings would be useful for future research aimed at developing new, specific inhibitors of trypanosomatid GAPDH.

## 4. *Plasmodium* spp.

The phylum Apicomplexa consists of a group of diverse protists sharing common morphological features. These parasites possess an apical complex, a suite of structures allowing them to invade the host cell. Most of them also have a relict plastid, the apicoplast, which is nonphotosynthetic but vital for their survival [108]. In humans, the medically important apicomplexans include *Plasmodium*, *Babesia*, *Cryptosporidium*, *Cyclospora*, *Isospora*, and *Toxoplasma* [109].

Malaria is a life-threatening mosquito-borne blood disease caused by species of the genus *Plasmodium*. Five species of *Plasmodium* have long been known to cause human malaria including *P. falciparum*, *P. malariae*, *P. ovale*, *P. vivax*, and *P. knowlesi* [110]. *P. falciparum* is the deadliest malaria parasite, causing the vast majority of malaria-associated mortality and morbidity [111]. Quinoline derivatives, antifolates, and artemisinin compounds are three main classes of antimalarial drugs [5]. Deplorably, efforts to control malaria have been thwarted by the emergence of drug resistance. Furthermore, the complicated life cycle of *Plasmodium* represents a major challenge for developing an effective vaccine [112].

### 4.1. Effects of Que on *Plasmodium berghei*

To test the effectiveness of antimalarial drugs, many researchers have used *P. berghei*-infected mouse models [113]. When Que (50 mg/kg body weight) was administered orally to mice infected with *P. berghei* once daily for three consecutive days, it effectively reduced the parasitemia by 52% and 44% on days 5 and 7, respectively [55]. In another study [56], daily intraperitoneal administration of Que to animals infected with *P. berghei* for four consecutive days not only curtailed the development of parasitemia but also prolonged the median survival time as compared to the nontreated infected group (Table 3). During a 30-day observation period, no major physical and behavioral changes (e.g., excess urination, diarrhea, lethargy, or locomotor activity decrements) were recorded in the noninfected mice receiving Que in comparison to the control group [56]. These studies suggest that Que is well tolerated in animals without any overt toxic effects.

Glycogen synthase kinase-3 (GSK3) is an evolutionary conserved serine/threonine protein kinase comprising two highly similar paralogs, namely, GSK3*α* and GSK3*β*. The former is thought to be regulated by phosphorylation at Ser-21 (inhibition) and Tyr-279 (activation), while the corresponding amino acids in the latter are Ser-9 and Tyr-216 [114]. With more than 40 known targets and over 500 proposed candidate substrates, GSK3 fulfills its role in numerous signaling pathways in the cell, such as inflammation, immune response, apoptosis, autophagy, and wound healing [115, 116]. GSK3 could therefore be considered as a potential target for therapeutic interventions. Interestingly, evidence suggests that Que plays a cytokine-modulatory role through GSK3*β* in *P. berghei*-infected mice [56]. When administered intraperitoneally, Que increased GSK3*β* (Ser-9) phosphorylation in the liver of *P. berghei* NK65-infected mice, thereby inhibiting GSK3*β* activity in their livers. Since GSK3*β* affects the immune responses by regulating cytokine production [115], the higher GSK3*β* activity in the liver of untreated NK65-infected animals may be associated with enhanced production of proinflammatory cytokines during inflammation [56]. Remarkably, exposure of *P. berghei* NK65-infected animals to Que resulted in not only a profound decrement in the levels of the proinflammatory cytokines such as TNF-*α* and IFN-*γ* but also a striking increase in the levels of the anti-inflammatory cytokines, especially IL-4 and IL-10. This modulation of cytokine balance may be a sequel of the suppressive effect of Que on GSK3*β*. GSK3 inhibition has also been proposed to drive the maturation and function of natural-killer (NK) cells [117]; hence, this could contribute to pathogen clearance.

Davoodi et al. [79] observed that Que nanophytosomes (NQ; 10 mg/kg) substantially reduced histopathological damage (e.g., Kupffer cell hyperplasia, hepatic necrosis, hemosiderosis, and periportal inflammation) and serum levels of both IL-1*β* and TNF-*α* in *P. berghei*-infected mice. Moreover, the best results were achieved when NQ (10 mg/kg) was applied in combination with hydroxychloroquine sulfate (2 mg/kg). Future research may consider using phytosomes in combination with various antimalarial drugs in clinical trials.

### 4.2. Effects of Que on *Plasmodium falciparum*

In a study conducted by Helgren et al. [118], different drug-resistant strains of *P. falciparum* were vulnerable to Que treatment (see Table 1). Similarly, several *P. falciparum* field isolates in Bangladesh were shown to be sensitive to Que [53]. Evidence also suggests that Que is highly specific for *Plasmodium* compared to mammalian cells [55, 56]. As regards combination therapy, the simultaneous use of Que, luteolin, and apigenin was shown to have an apparent additive inhibitory effect on the intraerythrocytic growth of the 7G8 strain [52]. In light of this observation, combinations of Que with other flavonoids merit further attention.

*Plasmodium* parasites acquire fatty acids by scavenging from the vertebrate host and the mosquito vector. They are also able to produce fatty acids de novo via the type two fatty acid synthesis (FAS-II) pathway [119]. The FAS-II pathway is localized to the apicoplast, a relict nonphotosynthetic plastid homologous to the chloroplasts of plants and algae [120]. This pathway catalyzes rounds of fatty acid elongation through the function of four important enzymes, namely, *β*-ketoacyl-acyl carrier protein (ACP) synthase I/II (FabB/F), *β*-ketoacyl-ACP reductase (FabG), *β*-hydroxyacyl-ACP dehydratase (FabZ), and enoyl-ACP reductase (FabI) [121]. The FAS-II pathway seems to be the ideal target since it has no homologs in humans. In a study conducted by Tasdemir et al. [51], a large library of flavonoids was tested against FabG, FabZ, and FabI. These three enzymes contribute to the fatty acid biosynthetic pathway in *P. falciparum*. Luteolin, Que, fisetin, and morin had inhibitory activities against all three enzymes. Structure–activity relationship analysis for the inhibition of FabG, FabZ, and FabI revealed that when the phenyl ring B is hydroxylated in two or three positions, the polyphenol becomes a very potent inhibitor of these enzymes, irrespective of the additional hydroxy group at C-3. Que exhibited strong inhibitory activity against the above-mentioned enzymes, with IC_50_ values ranging from 1.5 to 5.4 *μ*M [51]. Likewise, Sharma et al. [122] demonstrated that Que can reversibly inhibit *P. falciparum* enoyl-ACP reductase with Ki values in the nanomolar range. These findings suggest that inhibition of *P. falciparum* fatty acid biosynthesis is one of the possible mechanisms underlying the antiplasmodial effects of Que.

To avoid heme toxicity, *Plasmodium* is equipped with a unique detoxification system that converts soluble heme to an insoluble, nontoxic, crystalline pigment called hemozoin [123]. This process is facilitated by action of various proteins including heme detoxification protein (HDP) and histidine-rich proteins 2 and 3 (HRP-2 and HRP-3, respectively). Of these, HDP is the most potent in hemozoin formation and plays an indispensable role in parasite survival [124]. Interestingly, homologs of HDP have also been reported in other blood-feeding parasites such as *Theileria*, *Babesia*, and *Toxoplasma* [124]. Drugs that inhibit the conversion of heme to hemozoin have potent antimalarial activity. In this respect, numerous compounds such as azoles, isonitriles, quinolines, xanthones, and methylene blue have been shown to interfere with the free heme detoxification pathway, causing the pathogen to experience oxidative stress [123]. The crystal structure of the synthetic form of hemozoin is called *β*-hematin which can be used for in vitro assay analysis. Recently, in silico and in vitro studies have shown that Que can inhibit the formation of *β*-hematin [125, 126]. It has been suggested that the inhibition of *β*-hematin occurs due to the formation of a heme-Que complex [125]. However, further studies are needed to evaluate whether Que can inhibit the above-mentioned heme detoxification enzymes in *Plasmodium* species.

Oral pretreatment of Swiss albino mice with 50 mg/kg (body weight) of Que was shown to be effective in mitigating both hepatotoxicity and oxidative stress caused by chloroquine administration. Intriguingly, the same amount of Que resulted in a notable decrease in lipid peroxidation induced by chloroquine. Que was also capable of recouping the loss of glutathione content to almost normal levels in mice subjected to a high dose of chloroquine [127]. Glutathione plays a pivotal role in the antioxidative defense system of cells, protecting them against both oxidative damage and harmful xenobiotics [128]. Que can also augment the activity of several antioxidant enzymes (i.e., catalase, superoxide dismutase, glutathione reductase, and glutathione peroxidase) in chloroquine-treated animals. Moreover, pretreatment with Que was beneficial in relieving murine liver damage caused by chloroquine treatment [127]. Based on these data, one may suggest coadministration of Que and chloroquine for antimalarial treatment, especially when the latter is utilized as a long-term prophylactic therapy.

### 4.3. Effects of Que on *Plasmodium juxtanucleare*

Hemosporidians have been associated with reductions in the reproductive success and longevity in chronic infections and sometimes lead to disease outbreaks with high mortality rates in birds. *P. gallinaceum* and *P. juxtanucleare* are two species that naturally infect domestic chickens, causing “chicken malaria” [129]. In one study, chickens infected with *P. juxtanucleare* were immunocompromised by intramuscular injection of methylprednisolone before receiving 50 mg/kg (body weight) Que or chloroquine diphosphate by gavage for four consecutive days [80]. Interestingly, both treated groups exhibited a significant decrease in parasitemia as compared to the control group (*p* < 0.01) within the 30 days following the infection. Based on these preliminary in vivo data, it seems that Que has the potential to be used in animal husbandry and poultry farming.

## 5. *Toxoplasma gondii*

Humans and almost all warm-blooded animals are infected by *Toxoplasma gondii*, an obligate intracellular apicomplexan parasite. Members of the cat family are the only definitive hosts. The pathogen is transmitted to humans by eating undercooked meat from animals that have tissue cysts, contacting with infected cat feces, blood transfusion or organ transplantation, and vertical transmission during pregnancy [130]. The disease may be severe or life-threatening in immunocompromised patients. *T. gondii* typically forms tissue cysts in skeletal muscles, the myocardium, brains, and eyes in human hosts [131]. A combination of two antimicrobial agents is usually used to treat toxoplasmosis, inhibitors of dihydrofolate reductase (pyrimethamine and trimethoprim) and dihydropteroate synthase (sulfadoxine, sulfadiazine, and sulfamethoxazole). These two enzymes are sequentially involved in the folate pathway of nucleic acid synthesis [132].

### 5.1. Effects of Que on *Toxoplasma gondii*

In vitro experiments revealed an inhibitory effect of Que (100 *μ*M) on the expression of bradyzoite antigen [BAG1/hsp30 (BAG5)], which is triggered by either pH 8.1 or sodium nitroprusside. There was also a three- to fourfold decrease in the Hsp70 expression in *T. gondii* after being exposed to 100 *μ*M of Que at pH 8.1 [57]. From these data, it seems that Que could hinder stress-mediated induction of bradyzoite differentiation, possibly via inhibition of heat shock protein(s).

Using murine astrocytes to foster the development of the *T. gondii* cysts, Halonen et al. [58] examined the inhibitory effects of Que on cyst induction. When added to mature cysts (72 h old cysts developed in murine astrocytes), 12.5 *μ*M of Que increased the total number of cysts. However, high concentrations of Que (>25 *μ*M) decreased the total number of cysts as compared to the nontreated control, suggesting that the effects of Que on mature cysts are biphasic. Addition of Que to cultures at the time of infection resulted in increased cyst formation [58]. At higher concentrations of Que, the absolute number of cysts decreased while the percentage of cyst antigen-positive vacuoles increased. It has been noted that inhibition of *T. gondii* growth by Que is associated with the induction of cyst formation [58]. Taken together, these findings underscore the importance of both exposure time and concentration of Que in inhibiting cyst formation.

In one study, transfection of *T. gondii* tachyzoites with antisense oligonucleotides (AntiA, targeting the start codon of parasite Hsp70) and subsequent treatment with Que (50 *μ*M) was found to be more effective than Que (50 *μ*M) alone (without AntiA treatment) in reducing Hsp70 expression [59]. When RAW 264.7 cells were infected with treated virulent parasites to diminish Hsp70 expression, levels of iNOS message were substantially increased [59]. Likewise, virulent *T. gondii* strains expressing reduced levels of Hsp70 were not able to hinder the translocation of NF-*κ*B from the cytoplasm to the nucleus of murine splenocytes. By contrast, inhibition of Hsp70 expression in the avirulent strains of *T. gondii* had no significant impact on translocation of NF-*κ*B to the nucleus [59]. Since various Hsps and other proteins classically associated with the stress response have major roles in bradyzoite differentiation, they may serve as potential drug targets for toxoplasmosis [133].

The above-mentioned treatment (i.e., AntiA and Que) also effectively diminished Hsp70 expression in virulent (RH and ENT) and avirulent (ME49 and C) strains of *T. gondii* recovered from the peritoneal cavities of BALB/c mice [59]. In response to combination therapy, animals receiving the virulent *T. gondii* strains with reduced Hsp70 expression showed a lower splenic parasite burden than those infected with untreated virulent strains. Further preclinical studies are needed to fully investigate these events.

## 6. *Cryptosporidium* spp.

*Cryptosporidium* is an intracellular protozoan parasite belonging to the phylum Apicomplexa. The pathogen has emerged as one of the major causes of diarrheal diseases worldwide [134]. Mammalian cryptosporidiosis is most commonly caused by *C. parvum*. Nitazoxanide is the only Food and Drug Administration- (FDA-) approved drug for treating diarrhea caused by *Cryptosporidium* in individuals with healthy immune systems. Currently, there is no vaccine to prevent cryptosporidiosis [135].

### 6.1. Effects of Que on *Cryptosporidium parvum*

In a study conducted by Mead and McNair [60], Que was active against the *C. parvum* Iowa strain in the 5-32 *μ*M range. The study found that Que was much more toxic to *C. parvum* than the host cells (Table 2). Although the infection stimulated apoptosis of the host cells to some extent, no additional apoptosis was observed after treatment with Que [60]. Further research is needed to elucidate the anticryptosporidial mechanisms of Que.

## 7. *Eimeria* spp.

Coccidiosis is a widespread disease in livestock and poultry. It is caused by protozoan parasites of the apicomplexan genus *Eimeria*. The disease leads to high mortality, poor performance, and reduced productivity in domestic livestock [136]. All *Eimeria* species are monoxenous because their life cycle is completed within a single host. They are transmitted directly through the oral-fecal route. Infection occurs when oocysts are ingested. Sick animals often suffer from acute diarrhea with or without blood, decreased appetite, and depression [137]. Anticoccidial drugs belong to one of two categories: The first class is polyether antibiotics including monovalent ionophores (e.g., monensin, narasin, and salinomycin), monovalent glycosidic ionophores (e.g., maduramicin and semduramicin), and a divalent ionophore (e.g., lasalocid). The second class is synthetic compounds including inhibitors of parasite mitochondrial respiration (e.g., decoquinate and clopidol), inhibitors of the folic acid pathway (e.g., sulfonamides), competitive inhibitors of thiamine uptake (e.g., amprolium), and drugs with an unknown mode of action (e.g., diclazuril, halofuginone, nicarbazin, and robenidine). Combination drugs, consisting of either a synthetic compound and ionophore or two synthetic compounds, are also available [138].

### 7.1. Effects of Que on *Eimeria* spp.

Debbou-Iouknane et al. [61] conducted a study to determine the required time for maximum reduction of *Eimeria* spp. oocysts by measuring the kinetics of oocyst lysis in response to Que treatment. The oocysts, isolated from fresh feces of broiler chickens with coccidiosis, were a mixture of *E. acervulina*, *E. tenella*, *E. mitis*, *E. brunetti*, and *E. maxima*. The maximum observed decrease in the number of *Eimeria* spp. oocysts (45.4%) occurred after 8 h of incubation with Que (0.139 mg/mL). In another study [63], however, Que exhibited no growth inhibitory activity against *E. tenella* at concentrations up to 50 *μ*M.

Secondary messengers like Ca^2+^ regulate a multitude of cellular events in apicomplexan protozoa and serve as important intermediaries during their life cycle stages. In fact, changes in Ca^2+^ concentration play a pivotal role in protein secretion, motility, invasion, differentiation, and egress from infected cells [139]. Calcium-dependent protein kinases (CDPKs) are major effector molecules involved in calcium signaling pathways [140], thereby affecting above-mentioned physiological processes. Recently, molecular docking was used to screen several plant-based natural compounds for their potential inhibitory effects on *Eimeria* CDPK [141]. Que had the best interaction with *Eimeria* CDPK among the tested compounds (i.e., 6′-de-O-acetylcupacinoside, apigenin, artemisinin, cupacinoside, and rutin), with a binding energy of −7.04 kcal/mol. Additionally, the concentration needed to yield half-maximum inhibition in relation to the active site pocket interaction with Que was 6.94 *μ*M. Another study by Sun et al. [63] provided some molecular-level insights into the anti-*Eimeria* mechanism of action of Que. They observed that Que efficiently stymied the enzymatic activity of the hexokinase from *E. tenella* (EtHK) (see Table 1). Future studies should examine the interaction of EtHK with other flavonoids.

In one study, del Cacho et al. [62] used immunogold labelling of surface spreads of meiotic chromosomes from *E. tenella* oocysts to examine the effects of Que on the expression and ultrastructural localization of Hsp70. Immunoblot analysis of Hsp70 contents in Que-treated and nontreated oocysts revealed that the density of the bands decreased when the amount of Que was increased. Interestingly, exposure of *E. tenella* oocysts to Que caused a profound inhibition of Hsp70 synthesis (Table 1). Consequently, there was a failure to form synaptonemal complexes (SCs) or complete desynapsis and the inability to develop haploid sporozoites. The SC is a crucial and deeply conserved protein lattice that brings parental chromosomes into close proximity during the meiotic prophase, stabilizes their pairing, and regulates genetic recombination [142]. Considering its chaperon function, Hsp70 in *Eimeria* SCs may contribute to stabilization of structures essential for chromosomal paring and segregation. Overall, Que inhibits Hsp70 synthesis in *E. tenella* oocysts, prevents sporulation, and interrupts SC formation or desynapsis.

## 8. *Babesia* spp.

*Babesia* species are tick-borne apicomplexan pathogens that are obligate parasites of RBCs [143]. They reproduce asexually in the RBCs of mammalian hosts and sexually in their arthropod vectors. Over a hundred species are acknowledged to infect mammalian and avian hosts. Some species are known to be capable of causing infection in humans. Zoonotic species include, but not limited to, *B. microti*, *B. divergens*, and *B. duncani*. The epidemiology of human babesiosis is complex due to the diversity of *Babesia* species [144]. The existing armamentarium of chemotherapeutics for the treatment of human babesiosis relies principally upon atovaquone, azithromycin, clindamycin, and quinine [145].

### 8.1. Effects of Que on *Babesia* spp.

Que has been shown to act as a potent growth inhibitor of different *Babesia* species in vitro. In this context, IC_50_ values of Que for *B. bovis*, *B. bigemina*, and *B. caballi* were 8, 7, and 5 nM, respectively [64]. Even ten days after removal of Que, there was no indication of parasite recrudescence for *B. caballi*, *B. bovis*, and *B. bigemina* in mice treated with 50, 100, and 100 *μ*M Que, respectively. Microscopic analyses also revealed morphological changes in the intraerythrocytic forms of these parasites on day 4 after exposure to Que [64]. When injected intraperitoneally into mice, Que (14.5 mg/kg) considerably reduced parasitemia due to *B. microti* from days 4 to 8 postinoculation as compared to the control group [64]. Although some promising results were obtained in this study, further research is needed to replicate and extend these findings.

## 9. *Theileria* spp.

Similar to *Babesia*, *Theileria* is an apicomplexan tick-borne pathogen that infects a wide spectrum of domestic and wild animals [146]. Though *Babesia* species are primarily parasites of RBCs, *Theileria* species use both white blood cells and RBCs to complete their life cycle in a sequential manner. Diseases with the greatest economic impact on ruminants are East Coast fever (*T. parva*) and tropical theileriosis (*T. annulata*). *T. equi* causes piroplasmosis in horses, while *T. lestoquardi* infects sheep and goats. Only a single drug, buparvaquone, is available for the treatment of theileriosis [146].

### 9.1. Effects of Que on *Theileria equi*

One study reported that Que had considerable inhibitory effects on the in vitro growth of *T. equi*, with an IC_50_ value of 4 nM [64]. Importantly, the viability test demonstrated no sign of parasite recrudescence after the removal of Que (50 *μ*M) for 10 days. On day 4 after treatment with 25 *μ*M of Que, *T. equi* parasites appeared dot-shaped in RBCs [64]. Nevertheless, the precise mechanisms underlying these inhibitory effects should be elucidated in future studies.

## 10. *Trichomonas* spp.

*Trichomonas* is a genus of amitochondriate flagellated protists. Many species have symbiotic relationships with different animal hosts. There are four species of trichomonads found in humans including *T. vaginalis* (found in the urogenital tract), *T. tenax* (found in the oral cavity), *Pentatrichomonas hominis* (inhabits the intestinal tracts), and *Dientamoeba fragilis* (inhabits the intestinal tracts). Only *T. vaginalis* has a well-established pathogenic potential. *T. vaginalis* is the causative agent of the most frequent nonviral sexually transmitted infection in humans, trichomoniasis [147].

### 10.1. Effects of Que on *Trichomonas gallinarum*

*T. gallinae* and *T. gallinarum* are the most prevalent avian trichomonad pathogens [148]. There is only one study dealing with the antitrichomonad activity of Que against *T. gallinarum* [66]. In this respect, the minimal lethal concentration (MLC) of Que against *T. gallinarum* was reported to be equal to 0.121 *μ*g/mL after 24 h of incubation at 37°C. Therefore, Que appears to exert strong trichomonacidal activity in vitro. However, further research on various flavonoid classes is required to determine the most effective compounds and their optimal doses.

### 10.2. Effects of Que on *Trichomonas vaginalis*

One study revealed that 100 *μ*M of Que inhibited the growth of *T. vaginalis* strain G3 [65]. A related study showed that half-maximal growth inhibition of *T. vaginalis* strain GT15 occurred with only 21.17 ± 2.60 *μ*g/mL of Que [67]. Not only did 1000 *μ*g/mL of Que exhibit trivial hemolytic activity (0.7%) toward human RBCs, but it also imparted maximum erythroprotective effects (100%) when RBCs were exposed to 2,2′-azobis(2-methylpropionamidine) dihydrochloride (AAPH). As a water-soluble free radical initiator, AAPH can inflict oxidative damage on the RBC membrane, culminating in hemolysis [149]. These findings suggest that Que could behave as an erythroprotective agent by precluding radical-induced toxicity in human RBCs, as well as being an antitrichomonal substance.

### 10.3. Effects of Que on *Tritrichomonas foetus*

*T. foetus* is an obligate parasite of the bovine reproductive tract and intestinal tract of cats [150]. Que has been reported to be active against two strains of *T. foetus* [65]. Only 100 *μ*M of Que was sufficient to inhibit *T. foetus growth* (Table 1). A combination of low doses of metronidazole (a first-line drug) with bioactive plant compounds could act synergistically against trichomoniasis in both humans and animals [151].

## 11. *Entamoeba* spp.

Members of the genus *Entamoeba* are pseudopod-forming, nonflagellated protozoan parasites. Humans are home to multiple species, but not all of them are associated with diseases. The genus *Entamoeba* includes many species, six of which (*E. histolytica*, *E. dispar*, *E. moshkovskii*, *E. polecki*, *E. coli*, and *E. hartmanni*) reside in the human intestinal lumen. *E. histolytica* has long been recognized as a pathogenic amoeba, associated with intestinal (particularly amoebic dysentery) and extraintestinal infections [152].

### 11.1. Effects of Que on *Entamoeba histolytica*

The antiprotozoan activity of Que against *E. histolytica* has seldom been explored. For instance, one study demonstrated that the half-inhibitory concentration of Que against *E. histolytica* was 44.48 ± 3.92 *μ*g/mL [67]. Nonetheless, the molecular mechanisms responsible for the antiprotozoan activity of Que toward *E. histolytica* remain mysterious and need to be elucidated.

## 12. *Acanthamoeba* spp.

*Acanthamoeba* is a free-living amoeba that is ubiquitously distributed in the environment such as freshwater, seawater, chlorinated water from swimming pools, dental treatment units, contact lens cases, and solutions. It can cause sinusitis, skin lesions, vision-threatening keratitis, and granulomatous amoebic encephalitis [153].

### 12.1. Effects of Que on *Acanthamoeba castellanii*

*A. castellanii* is an important opportunistic pathogen which causes amoebic keratitis and occasionally granulomatous amoebic encephalitis [154]. In a recent study [68], Que or Que-conjugated silver nanoparticles (QAgNPs) were shown to exert potent in vitro amoebicidal activity against *A. castellanii* ATCC 50492 (Table 1). Que and QAgNPs (5 and 10 *μ*M, respectively) also exhibited minimal cytotoxicity in vitro against the human keratinocyte HaCaT cell line (Table 2). Unlike Que, QAgNPs effectively inhibited both encystation and excystation of *A. castellanii* after 72 h at 30°C, suggesting superior antiacanthamoebic activity of QAgNPs over Que. Nevertheless, the effectiveness of these compounds should be evaluated in an animal model of keratitis caused by *A. castellanii*.

## 13. Future Directions

Over the past decades, pharmaceutical companies have increasingly opted to exploit plant-based compounds for a variety of indications. Different side chains in flavonoids can have a significant impact on the activity of a particular flavonoid in the same target. To enhance their antiparasitic effects, several studies have focused on improving the structural features of Que and its derivatives through the process of acylation or alkylation of hydroxyl groups [27, 38]. Halogens can also be introduced into natural products or synthetic compounds to bolster their biological activities and physiochemical properties. For instance, halogenated derivatives of Que have been shown to possess more potent antioxidant [155], antitumor [156], and antidiabetic [157] properties compared with Que alone. Furthermore, the Que framework could be suitably modified by the insertion of sulfonate, prenyl, aminomethyl, and phenylethenyl appendages into its A- and B-rings to provide different derivatives. These new compounds were shown to have potent anticancer and hepatoprotective activities in vitro [158]. Future research should assess the antimicrobial activities of such novel derivatives against protozoan parasites. Additionally, it is worth noting that protozoan parasites have the ability to adapt and acquire resistance to numerous chemical compounds. Hence, it is imperative to devise strategies that involve a comprehensive understanding of the mechanisms underlying the action and resistance of newly discovered compounds, which have already advanced to later stages of clinical trials. This understanding would enable the design of alternative and safer molecules [5]. In future research, it is crucial to delve into the significance of interaction of Que with cellular components and its impact on the development of resistance in protozoan parasites.

Oral administration is by far the most convenient and preferred route of drug delivery. Poor solubility and instability of Que remain a major hurdle in achieving sufficient oral bioavailability. However, Que is a drug-like compound that conforms to Lipinski's rule of five without any violation, which indicates that a compound with ≤5 hydrogen-bond donors, ≤10 hydrogen-bond acceptors, molecular weight ≤ 500 Daltons, and calculated octanol-water partition coefficient (Clog P) ≤ 5 probably presents a high bioavailability [159]. A great deal of research has been directed toward enhancing the stability and bioavailability of Que. Thus far, various nutraceutical delivery systems such as polymeric micelles [160], nanoparticles [161], and phytosomes [162] have been developed for improving oral bioavailability of Que. Moreover, conjugation of Que with different amino acids such as L-glutamic acid, L-alanine, and L-aspartic acid results in increased solubility, stability, and cellular permeability as well as biological activity [163]. In this respect, the Que–glutamic acid conjugate exhibited a remarkable resistance to hydrolases, resulting in a much longer half-life (180 min). When compared with Que, the Que–aspartic acid and Que–glutamic acid conjugates demonstrated an enhanced intestinal permeability in Madin-Darby canine kidney (MDCK) cells [164]. Que-metal complexes (metals such as germanium, vanadium, and copper) also showed better pharmacokinetic properties and have been successfully tested for their activity on diverse human cancer cell lines [158]. Incorporation of Que into ointments, creams, gels, emulsions, microneedles, and various nanocarrier systems would be a promising approach in the management of localized cutaneous infections particularly leishmaniasis, ensuring a sustained release of Que at the desired site of action [165]. Despite this, a number of factors such as rational dosing, potential toxicity to human cells, healing kinetics, optimal timing of drug application, and accumulation in target sites ought to be considered in future studies.

**Figure 1:**
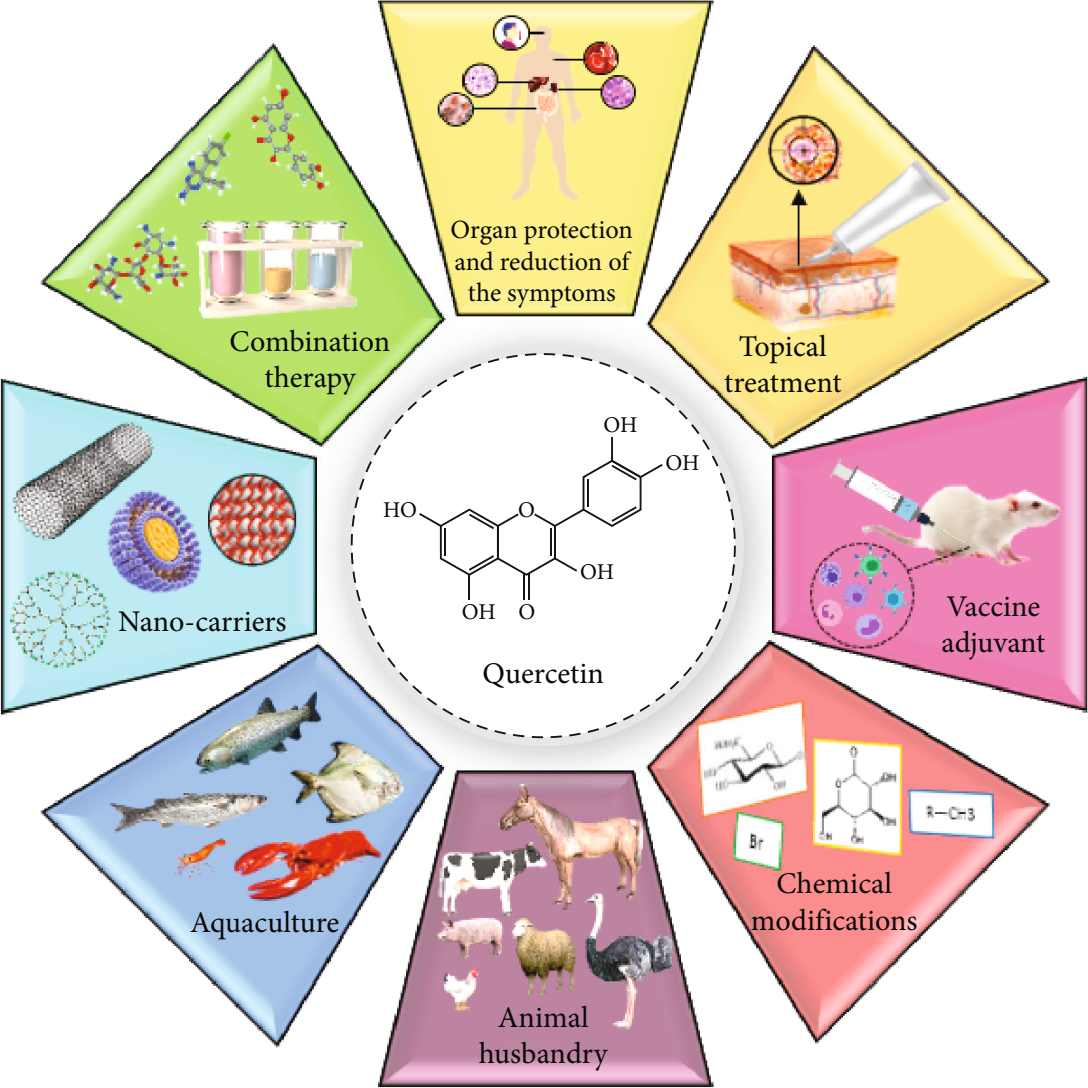
Schematic representation of various potential biomedical applications of Que for future studies on protozoan diseases.

Flavonoids could also act synergistically with the existing antiparasitic drugs. For example, Que, even at low concentrations, have already been demonstrated to boost therapeutic potential of antimalarial drugs such as chloroquine or artemisinin [52, 55, 79]. Moreover, the synergism between Que and antiparasitic drugs could diminish the development of drug-resistant pathogens in response to these compounds. Que can also be used to modulate the immune responses as well as reducing related pathology [166–168]. The protective ability of Que to ameliorate several pathological conditions has been well documented in the literature [169]. Another major feature of Que is its adjuvant potentiality for use in vaccines, which should be further explored for possible mechanistic aspects [170, 171]. Alongside human medicine, Que could be used in veterinary, animal husbandry, poultry farming, and aquaculture for both therapeutic and prophylactic purposes (Figure 1). Taken all together, more studies, especially well-designed clinical trials, are required to endorse the clinical efficacy of Que for the treatment of parasitic infections.

## 14. Conclusion

Hopes of eradicating the protozoan diseases such as malaria, leishmaniasis, and trypanosomiasis have been dashed due to the emergence of multi-drug-resistant strains together with the absence of effective vaccines. In recent years, Que has attracted a great deal of attention owing to its potential parasiticidal activity against a broad range of protozoan pathogens. A substantial body of scientific evidence has now provided unprecedented molecular-level insights into the antiprotozoan mechanisms of Que. Mitochondrial dysfunction, impairment in iron uptake, inhibition of certain enzymes involved in fatty acid synthesis and the glycolytic pathways, stimulation of apoptotic/necrotic cell death, and reduction in the expression of heat shock proteins are the major molecular mechanisms responsible for such inhibitory effects. Additionally, there is a burgeoning literature on protective effects of Que against parasitemia and histopathological damage in several animal models. All in all, Que could lay the foundation for a new generation of drugs that hold great promise for the treatment of infectious diseases.

## Figures and Tables

**Table 1:** List of in silico and in vitro studies dealing with antiprotozoan effects of quercetin. Developmental forms of tested protozoa, methods, and key findings of the relevant studies are also outlined.

Protozoan parasites	Identifiers	Developmental forms	Methods	Key findings	References
*Leishmania amazonensis*	MPRO/BR/72/M 1841	Promastigotes	MTT assay	(i) IC_50_ value of 123.5 *μ*g/mL after 24 h	[21]
	MHOM/BR/LTB0016	Promastigotes	Counting the number of promastigotes, Alamar Blue assay, determination of ΔΨ_m_, and measurement of ROS levels	(i) Dose- and time-dependent reduction in promastigote viability, with an IC_50_ value of 31.4 *μ*M after 48 h (ii) Dose-dependent induction of ROS production (iii) Depolarization of ΔΨ_m_	[22]
	NA	NA	UV-VIS spectrophotometry, evaluation of arginase inhibition, and molecular docking	(i) Inhibiting 50% of the catalytic activity of arginase at 4.3 *μ*M (ii) Interaction of Que with the substrate L-arginine and Mn^2+^ at pH 9.6 (iii) Interaction of the catechol group of Que with Asp-129	[23]
	MHOM/BR/75/LTB0016	Intracellular amastigotes	Counting the number of intracellular amastigotes and measurement of ROS levels	(i) Dose-dependent reduction in intracellular amastigotes, with an IC_50_ value of 3.4 *μ*M after 72 h (ii) Dose-dependent induction of ROS production	[20]
	MHOM/77BR/LTB0016	Promastigotes and intracellular amastigotes	MTT assay and counting the number of intercellular amastigotes	(i) Que: IC_50_ ± SD of 0.2 ± 0.06 *μ*g/mL (0.7 ± 0.2 *μ*M) and 1.3 ± 0.1 *μ*g/mL (4.3 ± 0.3 *μ*M) against promastigotes and amastigotes, respectively (ii) Superiority of Que over meglumine antimoniate (Glucantime®) in inhibiting leishmanial growth (iii) Glucantime®: IC_50_ ± SD of >1500 *μ*g/mL (>2954 *μ*M) and 11.0 ± 3.4 *μ*g/mL (21.7 ± 6.2 *μ*M) against promastigotes and amastigotes, respectively (iv) SI value of 10 for Que	[19]
	clon 1: Lma, MHOM/BR/76/LTB-012	Promastigotes	XTT assay	(i) IC_50_ ± SD of 20 ± 0.77 *μ*g/mL (66 *μ*M) (ii) Lower inhibitory activity of Que in comparison to AmB (IC_50_ ± SD: 0.21 ± 0.06 *μ*g/mL)	[24]

*Leishmania braziliensis*	MHOM/BR/1975/M2904	Promastigotes	Counting the number of promastigotes	(i) IC_50_ value of 30.49 *μ*M (ii) Lower inhibitory activity of Que in comparison to Glucantime® (25.61 *μ*M) and SSG (9.56 *μ*M)	[25]
	Strand M2904 C192 RJA	Promastigotes	XTT assay	(i) IC_50_ ± SD of 41 ± 2.7 *μ*g/mL (136 *μ*M) (ii) Lower inhibitory activity of Que in comparison to AmB (IC_50_ ± SD: 0.08 ± 0.04 *μ*g/mL)	[24]
	MHOM/BR/1987/M11272	Promastigotes and intracellular amastigotes	Counting the number of promastigotes and amastigote-infected M*φ*, measurement of ROS levels, determination of the cell death mode, evaluation of promastigote membrane integrity, promastigote recovery assay, cytokine assay, determination of nitrite levels, determination of labile iron concentration and total bound iron, immunocytochemical assays, and real-time PCR	(i) Substantial killing (>50%) of promastigotes (at Que concentrations of 48, 70, 96, and 192 *μ*M) after 24 h (ii) Increasing ROS production (iii) Induction of apoptosis/necrosis (iv) Decreasing the percentages of infected M*φ*, the number of amastigotes per M*φ*, and the recovered promastigotes (v) Reduction in TNF-*α* levels (vi) Increasing IL-10 synthesis without modulating NO production (vii) Modulation of labile iron pool in infected M*φ* (viii) Upregulation of Nrf2/HO-1 expression	[26]
	MHOM/BR88/BA-3456	Promastigotes	Alamar Blue assay	(i) IC_50_ value of >100 *μ*M (ii) Lower inhibitory activity of Que in comparison to AmB (IC_50_ ± SD: 1.1 ± 0.1 *μ*M)	[27]
	MCAN/BR/98/R619	Promastigotes and intracellular amastigotes	Counting the number of promastigotes and intracellular amastigotes, measurement of ROS/H_2_O_2_ levels, and evaluation of NO production	(i) Dose-dependent reduction in the number of promastigotes (IC_50_ ± SD of 25 ± 0.7 *μ*M, after 96 h) and amastigotes (IC_50_ ± SD of 21 ± 2.5 *μ*M, after 48 h) (ii) SI value of 22 for Que (iii) Lower inhibitory activity of Que in comparison to miltefosine (iv) Increasing ROS/H_2_O_2_ production without altering NO production by M*φ*	[28]

*Leishmania donovani*	AG83 strain	Promastigotes and intracellular amastigotes	Counting the number of promastigotes and intracellular amastigotes, DNA cleavage analysis, decatenation assay, flow cytometric analysis of cell cycle, and determination of the cell death mode	(i) Dose-dependent reduction in promastigote viability, with an IC_50_ value of 45.5 *μ*M after 24 h (ii) Reduction in the number of amastigotes per M*φ* (iii) Induction of morphological changes in promastigotes (iv) Induction of topoisomerase II-mediated kinetoplast DNA cleavage (v) Arresting cell cycle progression in promastigotes	[29]
	MHOM/ET/67/L82	Promastigotes	Counting the number of promastigotes	(i) Reduction in promastigote viability, with IC_50_ ± SEM of 63.8 ± 1.48 *μ*M (ii) Lower inhibitory activity of Que in comparison to PTM (IC_50_ ± SEM: 0.41 ± 0.18 *μ*M)	[30]
	NA	NA	Plasmid relaxation assay, duplex oligonucleotide cleavage assay, fluorescence binding assay, Que–DNA intercalation assay, single turnover cleavage, and religation experiment	(i) Inhibition of LdTOP1LS relaxation activity in comparison to the control enzyme, under both preincubation and simultaneous conditions (ii) Increasing cleavage by 30–36% with respect to the extent of cleavable complex formed without the drug (iii) Stabilization of the covalent complex formed between 25-mer duplex DNA and LdTOP1LS (iv) Acting reversibly against LdTOP1LS (v) Intercalation into DNA at a very high concentration (300 *μ*M) without binding to the minor grove (vi) Inhibition of the religation step using Que	[31]
	MHOM/ET/67/L82	Axenic amastigotes	Alamar Blue assay	(i) IC_50_ value of 1 *μ*g/mL after 72 h (ii) Lower inhibitory activity of Que in comparison to miltefosine (IC_50_ value of 0.34 *μ*g/mL)	[32]
	MHOM/IN/80/DD8	Promastigotes and intracellular amastigotes	Plasmid relaxation assay, evaluation of acid phosphatase activity, and measurement of luciferase activity	(i) Higher sensitivity of *Leishmania* topoisomerase I to Que treatment compared with that of human monocyte (2.7-fold; based on 50% inhibition of DNA relaxation activity) (ii) Higher sensitivity of amastigotes to Que treatment compared with promastigotes	[33]
	MHOM/IN/1983/AG83 and SSG- and PMM-resistant strains	Axenic and intracellular amastigotes	Counting the number of axenic and intracellular amastigotes (in M*φ*)	(i) Que against axenic amastigotes: IC_50_ ± SD of 34 ± 3, 150 ± 25, and 75 ± 10 *μ*M for WT, SSG-resistant, and PMM-resistant strains, respectively (ii) QAunp against axenic amastigotes: IC_50_ ± SD of 15 ± 3, 40 ± 8, and 30 ± 6 for WT, SSG-resistant, and PMM-resistant strains, respectively (iii) Que against intracellular amastigotes: IC_50_ ± SD of 30 ± 6, 120 ± 14, and 60 ± 10 *μ*M for WT, SSG-resistant, and PMM-resistant strains, respectively (iv) QAunp against intracellular amastigotes: IC_50_ ± SD of 10 ± 2, 35 ± 6, and 18 ± 3 *μ*M for WT, SSG-resistant, and PMM-resistant strains, respectively (v) QAunp showed the highest SI value among tested agents (i.e., AmB, SSG, PMM, and Que)	[34]
	LV82 strain	Promastigotes and intracellular amastigotes	Alamar Blue assay, counting the number of intracellular amastigotes, flow cytometric analysis of parasitic loads in M*φ*, determination of the cell death mode, comet assay, quantification of NO production by infected M*φ*, TEM, and real-time PCR	(i) Dose-dependent reduction in promastigote viability, with an IC_50_ value of 84.65 *μ*g/mL after 72 h (ii) Reduction in amastigote viability in the presence of Que (iii) Lower killing activity of Que against intracellular amastigotes in comparison to SSG at concentrations ranging from 15.62 to 500 *μ*g/mL (iv) Direct intercalation of Que into DNA as well as inducing double-stranded DNA damage in promastigotes (v) Substantial production of NO in infected M*φ* after 72 h exposure to Que (vi) Induction of ultrastructural changes in promastigotes after 72 h exposure to Que (vii) Downregulation of the expression levels of *Try-R* and *Try-S*	[35]

*Leishmania infantum*	NM	Promastigotes and intracellular amastigotes	Counting the number of promastigotes and intracellular amastigotes	(i) Increasing antileishmanial activity of Que by PLC nanoencapsulation (ii) Dose-dependent reduction in the number of promastigotes and intracellular amastigotes after 192 h exposure to either Que or QPNPs (iii) Que: IC_50_ values of 149 and 300 *μ*g/mL against promastigotes and intracellular amastigotes, respectively (iv) QPNPs: IC_50_ values of 86 and 144 *μ*g/mL against promastigotes and intracellular amastigotes, respectively	[36]
	NA	NA	Evaluation of *L. infantum* arginase inhibition	(i) Potent inhibition of the enzyme activity (67.05 ± 10.36%) using 100 *μ*M of Que	[37]

*Leishmania major*	MHOM/DZ/2000/LIPA1126	Promastigotes	MTT assay, determination of the cell death mode, fluorescence microscopy, and evaluation of protease activity in promastigotes	(i) Dose-dependent reduction in promastigote viability after 24 h (ii) Induction of morphological changes (iii) Induction of necrosis and apoptosis via caspase-independent pathways	[10]
	NM	Promastigotes	Counting the number of promastigotes	(i) Dose-dependent reduction in promastigote viability, with IC_50_ ± SD of 2.5 ± 0.92 *μ*M after 48 h (ii) Superiority of Que over Glucantime® in killing promastigotes at all concentrations tested	[38]
	NM	Promastigotes, intracellular amastigotes, and axenic amastigotes	Alamar Blue assay	(i) IC_50_ and IC_90_ values of >10 *μ*M against promastigotes, amastigotes inside THP-1 cells, and axenic amastigotes (ii) Lower inhibitory activity of Que in comparison to PTM and AmB	[39]
	MHOM/IR/75/ER	Promastigotes	MTT assay	(i) Dose- and time-dependent reduction in promastigote viability, with an IC_50_ value of 16 *μ*M after 24 h	[40]
	MRHO/IR/75/ER	Promastigotes and intracellular amastigotes	MTT assay and counting the number of intracellular amastigotes	(i) Dose-dependent reduction in the number of promastigotes (IC_50_ ± SEM of 0.27 ± 0.08 *μ*M) and intracellular amastigotes (IC_50_ ± SEM of 0.85 ± 0.30 *μ*M) (ii) Superiority of Que over Glucantime® in reducing the number of promastigotes and amastigotes	[41]
	MRHO/IR/75/ER	Promastigotes	Counting the number of promastigotes and MTT assay	(i) Time-dependent cytotoxicity of Que and AgNPs/Que against promastigotes (ii) IC_50_ values of 150 and 125 *μ*g/mL for Que and AgNPs/Que, respectively (iii) Growth inhibitory and killing activities against promastigotes: AgNPs/Que > Que > Glucantime®	[42]

*Leishmania tropica*	NM	Promastigotes and axenic amastigotes	MTT assay and DNA degradation analysis	(i) Dose- and time-dependent activity against the parasite (ii) Against promastigotes: IC_50_ values of 232.4, 230.9, and 182.3 *μ*g/mL after 24, 48, and 72 h, respectively (iii) Against amastigotes: IC_50_ values of 207.5, 163.5, and 137.4 *μ*g/mL after 24, 48, and 72 h, respectively (iv) Lower inhibitory activity of Que in comparison to AmB (i.e., complete growth inhibition) at all concentrations tested (v) Induction of DNA fragmentation in promastigotes after 72 h	[43]

*Trypanosoma brucei brucei*	S427 strain	Bloodstream trypomastigote forms	Counting the number of live parasites	(i) Reduction in trypomastigote viability, with IC_50_ ± SEM of 13.2 ± 1.1 *μ*M (ii) Lower inhibitory activity of Que in comparison to PTM (IC_50_ ± SEM of 3.4 × 10^−4^ ± 4 × 10^−5^ *μ*M)	[30]
	29–13 strain	Bloodstream and procyclic forms	Measurement of TbHK1 activity, tryptophan quenching assay of TbHK1, fluorescence microscopy, RNAi studies, and evaluation of TbHK1 overexpression	(i) Acting as a mixed-type inhibitor of TbHK1 (IC_50_ = 4.1 ± 0.8 *μ*M) (ii) Quenching of Trp-177 emission in TbHK1 (iii) Accumulation of Que in glycosomes (iv) Increasing sensitivity to Que by RNAi-mediated silencing of TbHK1 (v) Increasing protection against Que by overexpressing TbHK1 in the parasites	[44]
	Lister 427 strain	Bloodstream trypomastigote forms	Growth curve analysis, assessment of TbHsp70.c-Tbj2 ATPase activity, and molecular docking	(i) Inhibition of the parasite growth (ii) Substantial inhibition (9.3-fold) of Tbj2-stimulated ATPase activity of TbHsp70.c (iii) Binding to the nucleotide binding pocket of TbHsp70.c as well as forming hydrogen bonds with residues T-10, T-11, K-68, and G-202 (iv) Hydrophobic interactions between Que and TbHsp70.c (a docking score of −9.5 kcal/mol)	[45]
	NM	Trypomastigote forms	Alamar Blue assay and molecular docking	(i) IC_50_ and IC_90_ values of 7.52 and 9.76 *μ*M, respectively (ii) Lower inhibitory activity of Que in comparison to PTM (with IC_50_ and IC_90_ values of 0.001 and 0.002 *μ*M, respectively) (iii) Interactions between Que and TbHK1 (with a docking score of −6.62 kcal/mol) (iv) Induction of conformational changes in TbHK1 upon Que binding	[39]

*Trypanosoma brucei gambiense*	FéoITMAP/1893 and OK/ITMAP/1841	Bloodstream trypomastigote forms	Trypanolysis assay, determination of the cell death mode, ELISA (measurement of TNF-*α* levels), and quantification of NO production	(i) Dose-dependent reduction in the parasite viability, with an IC_50_ value of 10 *μ*M after 24 h (ii) Induction of dose- and time-dependent apoptosis in *T. b. gambiense* (iii) Reducing the release of proinflammatory mediators, such as TNF-*α* and NO derivatives from activated M*φ*	[46]

*Trypanosoma brucei rhodesiense*	STIB 900 strain	Bloodstream trypomastigote forms	Alamar Blue assay	(i) IC_50_ value of 8.3 *μ*g/mL after 72 h (ii) Lower inhibitory activity of Que in comparison to melarsoprol (IC_50_ value of 0.0026 *μ*g/mL)	[32]

*Trypanosoma cruzi*	Tulahuen strain	Epimastigotes	Measurement of mitochondrial ATPase activity	(i) Inhibition of both soluble and membrane-bound ATPase after incubation with Que (10 *μ*g/mL) for 10 min	[47]
	Y strain	Bloodstream trypomastigote forms	Counting the number of live parasites	(i) Exerting trypanocidal activity, with an IC_50_ value of 186.8 *μ*M after 24 h	[48]
	Y strain	Bloodstream trypomastigote forms	Counting the number of live parasites	(i) Reduction in trypomastigote viability, with an IC_50_ value of 233.20 *μ*M after 24 h	[21]
	Tulahuen strain C2C4 containing the *β*-galactosidase (*LacZ*) gene	Culture-derived trypomastigotes	CPRG-based colorimetric assay	(i) IC_50_ value of >30 *μ*g/mL after 96 h (ii) Lower inhibitory activity of Que in comparison to benznidazole (IC_50_ value of 0.328 *μ*g/mL)	[32]
	NA	NA	Evaluation of *T. cruzi* GAPDH inhibition by Que, nonspecific inhibition assays (in the presence of Triton X-100), and molecular docking	(i) IC_50_ value of 142 *μ*M for *T. cruzi* GAPDH (ii) Specific inhibition of *T. cruzi* GAPDH (this inhibition was not reversed/affected in the presence of Triton X-100) (iii) Interaction of Que with the active site of *T. cruzi* GAPDH (with a docking energy of −7.43 kcal/mol) (iv) Stabilization of Que by two hydrogen bonds with Ala-198 and Pro-253	[49]

*Plasmodium falciparum*	FCMSU1/Sudan and FCR_3TC_ strains	Intraerythrocytic parasites	[^3^H]-hypoxanthine incorporation assay	(i) Inhibition of the parasite growth (after 48 h), with IC_50_ and IC_90_ values of 6.9 and 11.4 *μ*g/mL, respectively	[50]
	K1 strain	Intraerythrocytic parasites	[^3^H]-hypoxanthine incorporation assay	(i) Reduction in promastigote viability, with IC_50_ ± SEM of 14.2 ± 2.2 *μ*M (ii) Lower inhibitory activity of Que in comparison to CHQ (IC_50_ ± SEM of 0.59 ± 0.10 *μ*g/mL)	[30]
	A CHQ-sensitive strain (NF54) and a CHQ-resistant strain (K1)	Intraerythrocytic parasites	[^3^H]-hypoxanthine incorporation assay and evaluation of inhibition of *P. falciparum* FabG, FabZ, and FabI enzymes by Que	(i) Inhibition of the parasite growth, with IC_50_ values of 10 and 8.9 *μ*M for NF54 and K1, respectively (ii) Inhibition of three enzymes involved in the fatty acid biosynthesis: IC_50_ values of 5.4, 1.5, and 1.5 *μ*M for FabG, FabZ, and FabI, respectively	[51]
	A CHQ-sensitive strain (3D7) and a CHQ-resistant strain (7G8)	Intraerythrocytic parasites	[^3^H]-hypoxanthine incorporation assay	(i) Inhibition of the parasite growth (after 96 h), with IC_50_ ± SEM of 15 ± 5 and 14 ± 1 *μ*M for 3D7 and 7G8, respectively (ii) Lower inhibitory activity of Que in comparison to CHQ (IC_50_ ± SEM of 0.006 ± 0.0003 and 0.084 ± 0.026 *μ*M for 3D7 and 7G8, respectively)	[52]
	Several field isolates from Bangladesh as well as culture-adapted 3D7 and K1 clones	Intraerythrocytic parasites	HRP-2 ELISA	(i) Inhibition of the parasite growth (after 72 h), with IC_50_ ± SD of 14.7 ± 12.62, 4.11 ± 2.05, and 2.94 ± 2.41 *μ*M for the field isolates, 3D7, and K1, respectively (ii) Lower inhibitory activity of Que in comparison to DHA (IC_50_ ± SD of 0.006 ± 0.012, 0.001 ± 0.003, and 0.001 ± 0.002 *μ*M for the field isolates, 3D7, and K1, respectively)	[53]
	FcB1 strain	Intraerythrocytic parasites	[^3^H]-hypoxanthine incorporation assay	(i) Inhibition of the parasite growth, with an IC_50_ value of 9 *μ*g/mL (ii) Lower inhibitory activity of Que in comparison to CHQ (IC_50_ ± SD of 0.0720 ± 0.0150 *μ*g/mL)	[54]
	CHQ-resistant clone W2	Intraerythrocytic parasites	HRP-2 ELISA	(i) Inhibition of the parasite growth, with IC_50_ ± SD of 13.0 ± 8.4 *μ*g/mL (ii) Lower inhibitory activity of Que in comparison to CHQ (IC_50_ ± SD of 0.175 ± 0.02 *μ*g/mL)	[55]
	3D7 strain	Intraerythrocytic parasites	*Plasmodium* lactate dehydrogenase assay	(i) Inhibition of the parasite growth, with IC_50_ ± SD of 19.31 ± 1.26 *μ*M (ii) Lower inhibitory activity of Que in comparison to CHQ (IC_50_ ± SD of 0.0023 ± 0.0001 *μ*M)	[56]

*Toxoplasma gondii*	ME49 strain	Bradyzoites	Western blotting, immunofluorescence microscopy, and immunoelectron microscopy	(i) Inhibition of SNP- and pH 8.1-associated induction of bradyzoite antigens using 100 *μ*M of Que (ii) Decreasing the total number of bradyzoite antigen-positive vacuoles at either pH 7.1 or 8.1 using 100 *μ*M of Que (iii) Reduction in Hsp70 expression in pH 8.1-treated *T. gondii*	[57]
	ME49 strain	Tachyzoites	^3^H-uracil uptake assay and immunofluorescence microscopy	(i) Association between inhibition of *T. gondii* growth and cyst induction with Que (ii) When added to mature cysts: 12.5 *μ*M of Que increased the total number of cysts; however, >25 *μ*M of Que decreased the total number of cysts (iii) When added to the culture at the time of infection, ≤12.5 *μ*M of Que increased cyst formation. At higher concentrations of Que (≥25 *μ*M), while the absolute cyst number decreased, the percentage of cyst wall-positive vacuoles increased	[58]
	Two virulent (RH, ENT) and two avirulent (ME49, C) strains	Tachyzoites	Western blotting, immunocytochemical analysis, iNOS analysis (by semiquantitative PCR), NO analysis (by colorimetric assay), and NF-*κ*B analysis (by fluorescent microscopy)	(i) Reduction in Hsp70 expression in *T. gondii* using Que with or without AntiA pretransfection (77% vs. 50%) (ii) Significant enhancement of expression of the BAG1 bradyzoite marker in the virulent strains with reduced Hsp70 expression (i.e., those treated with a combination of AntiA and Que) compared with the untreated virulent strains (*p* < 0.05) (iii) Elevation of NO production in RAW 264.7 cells infected with the virulent parasites expressing reduced levels of Hsp70 (due to combination treatment) (iv) Inability of the virulent parasites with reduced Hsp70 expression levels to inhibit translocation of NF-*κ*B from the cytoplasm to the nucleus of murine splenocytes	[59]

*Cryptosporidium parvum*	Iowa strain	Intracellular parasites	Immunofluorescence labeling of parasites using C3C3-FITC, TUNEL assay, and caspase-3/7 fluorometric assay	(i) Inhibition of the parasite replication, with an IC_50_ value of 30 *μ*M after 48 h (ii) Lower inhibitory activity of Que in comparison to trifluralin (IC_50_ value of 1 *μ*M) (iii) No further increase in apoptosis following treatment with Que	[60]

*Eimeria spp.*	An oocyst mixture (*E. acervulina*, *E. tenella*, *E. mitis*, *E. brunetti*, and *E. maxima*) isolated from fresh feces of broiler chickens	Oocysts	Kinetic studies of oocyst lysis (measurement of absorbance at 273 nm)	(i) Maximum decrease (45.38%) in the number of *Eimeria* spp. oocysts after 8 h incubation	[61]

*Eimeria tenella*	NM	Oocysts	SC study, TEM, Western blotting, and evaluation of oocyst sporulation	(i) Inability of meiotic chromosomes from oocysts treated with Que (100 *μ*M) to form SCs (ii) Breakage of one of the homologs in most of the bivalents from the oocysts incubated with Que (50 *μ*M) (iii) Reduction in Hsp70 expression (iv) Inhibition of oocyst sporulation	[62]
	Guangdong strain	Oocysts	Quantitative RT-PCR and measurement of EtHK activity	(i) No growth inhibitory activity against *E. tenella* at ≤50 *μ*M of Que (ii) Inhibition of enzymatic activity of recombinant EtHK, with IC_50_ ± SEM of 6.52 ± 1.23 *μ*M	[63]

*Babesia bovis*	Texas strain	Intraerythrocytic parasites	Microscopic assessment of parasites	(i) Inhibition of the parasite growth (after 96 h), with IC_50_ ± SD of 8 ± 2 nM (ii) Superiority of Que over DIZE (IC_50_ ± SD of 300 ± 30 nM) (iii) Induction of morphological changes in the parasite (iv) No parasite recrudescence after 10 days from the removal of Que (100 *μ*M)	[64]

*Babesia bigemina*	Argentina strain	Intraerythrocytic parasites	Microscopic assessment of parasites	(i) Inhibition of the parasite growth (after 96 h), with IC_50_ ± SD of 7 ± 1 nM (ii) Superiority of Que over DIZE (IC_50_ ± SD of 190 ± 20 nM) (iii) Induction of morphological changes in the parasite (iv) No parasite recrudescence after 10 days from the removal of Que (100 *μ*M)	[64]

*Babesia caballi*	A horse-isolated strain	Intraerythrocytic parasites	Microscopic assessment of parasites	(i) Inhibition of the parasite growth (after 96 h), with IC_50_ ± SD of 5 ± 1 nM (ii) Superiority of Que over DIZE (IC_50_ ± SD of 10 ± 2 nM) (iii) Induction of morphological changes in the parasite (iv) No parasite recrudescence after 10 days from the removal of Que (50 *μ*M)	[64]

*Theileria equi*	USDA strain	Intraerythrocytic parasites	Microscopic assessment of parasites	(i) Inhibition of the parasite growth (after 96 h), with IC_50_ ± SD of 4 ± 0.5 nM (ii) Superiority of Que over DIZE (IC_50_ ± SD of 710 ± 15 nM) (iii) Induction of morphological changes in the parasite (iv) No parasite regrowth after 10 days from the removal of Que (50 *μ*M)	[64]

*Tritrichomonas foetus*	Feline strain C1 and bovine strain D1	Trophozoites	Counting the number of live parasites	(i) Inhibition of the parasite growth (strain C1: 8.5% ± 2.2, and strain D1: 18.9% ± 1.9) in the presence of Que (100 *μ*M)	[65]

*Trichomonas gallinarum*	NM	Trophozoites	Microplate method (calculation of MLC)	(i) MLC value of 0.121 *μ*g/mL after 24 h	[66]

*Trichomonas vaginalis*	G3 strain	Trophozoites	Counting the number of live parasites	(i) Inhibition of the parasite growth (45.6% ± 1.6) in the presence of Que (100 *μ*M)	[65]
	GT15 strain	Trophozoites	Counting the number of live parasites	(i) Inhibition of the parasite growth (after 24 h), with IC_50_ ± SD of 21.17 ± 2.60 *μ*g/mL (ii) Lower inhibitory activity of Que in comparison to MNZ (IC_50_ ± SD of 0.09 ± 0.01 *μ*g/mL)	[67]

*Entamoeba histolytica*	HM1-IMSS strain	Trophozoites	Counting the number of live parasites	(i) Inhibition of the parasite growth (after 72 h), with IC_50_ ± SD of 44.48 ± 3.92 *μ*g/mL (ii) Lower inhibitory activity of Que in comparison to MNZ (IC_50_ ± SD of 0.17 ± 0.03 *μ*g/mL)	[67]

*Acanthamoeba castellanii*	ATCC 50492	Trophozoites and cysts	Counting the number of live parasites, encystation assay, and excystation assay	(i) Significant reduction in the amoebal viability using either Que (5 and 10 *μ*M) or QAgNPs (5 and 10 *μ*M) after 24 h when compared to the negative control (*p* < 0.05) (ii) Superiority of QAgNPs over Que in killing amoebae (iii) Significant inhibition of amoebic encystation by QAgNPs (10 *μ*M) after 72 h as compared to Que and AgNPs alone (*p* < 0.05) (iv) Significant inhibition of amoebic excystation by QAgNPs (5 *μ*M) after 72 h as compared to Que and AgNPs alone (*p* < 0.05) (v) Chlorhexidine (50 or 100 *μ*M) was shown to be the most effective treatment (100% reduction in the amoebal viability and inhibition of amoebic encystation)	[68]

AgNPs: silver nanoparticles; AmB: amphotericin B; AntiA: antisense oligonucleotides targeting the *T. gondii* Hsp70 ATG start codon; C3C3-FITC: anti-*C. parvum* fluorescein-labeled monoclonal antibody; CHQ: chloroquine; CPRG: chlorophenol red-*β*-D-glucopyranoside; ΔΨ_*m*_: mitochondrial membrane potential; DHA: dihydroartemisinin; DIZE: diminazene aceturate; ELISA: enzyme-linked immunosorbent assay; GAPDH: glyceraldehyde-3-phosphate dehydrogenase; HO-1: heme oxygenase-1; H_2_O_2_: hydrogen peroxide; HRP-2 ELISA: histidine-rich protein 2 enzyme-linked immunosorbent assay; Hsp70: heat shock protein 70; IC_50_: the concentration required to give 50% inhibition; IL-10: interleukin 10; iNOS: inducible nitric oxide synthase; LdTOP1LS: bisubunit topoisomerase I of *L. donovani*; M*φ*: macrophages; MLC: the lowest concentration of the compound at which no motile organism is observed; MNZ: metronidazole; NA: not applicable; NF-*κ*B: nuclear factor kappa B; NM: not mentioned; NO: nitric oxide; Nrf2: nuclear factor erythroid 2-related factor 2; PMM: paromomycin; PTM: pentamidine; QAgNPs: quercetin-conjugated silver nanoparticles; QAunp: quercetin-conjugated gold nanoparticles, QPNPs: quercetin-loaded poly-*ε*-caprolactone nanoparticles; Que: quercetin; RNAi: RNA interference; ROS: reactive oxygen species; SC: synaptonemal complex; SD: standard deviation; SEM: standard error of the mean; SI: selectivity index (CC_50_ for macrophages/IC_50_ for intracellular amastigotes); SNP: sodium nitroprusside; SSG: sodium stibogluconate (Pentostam®); TbHK1: *T. brucei* hexokinase 1; Tbj2: *Trypanosoma brucei* J protein 2; TEM: transmission electron microscopy; THP-1: human monocytic leukemia cell line; TNF-*α*: tumor necrosis factor-alpha; Try-R: trypanothione reductase; Try-S: trypanothione synthetase; TUNEL: terminal deoxynucleotidyl transferase- (TdT-) mediated dUTP nick end labeling; USDA: the United States Department of Agriculture; UV-VIS: ultraviolet-visible; WT: wild type.

**Table 2:** In vitro cytotoxic activities of quercetin against mammalian cells.

Cells	Methods	Incubation time	CC_50_	References
Human Chang liver cell line	MTT assay	48 h	868.22 ± 3.81 *μ*M	[56]
Human fetal lung fibroblast cell line MRC-5	Alamar Blue assay	72 h	>80 *μ*M	[27]
Human hepatocellular carcinoma cell line HepG2	Alamar Blue assay	72 h	>80 *μ*M	[27]
Human intestinal adenocarcinoma cell line HCT-8	MTT assay	48 h	>100 *μ*M	[60]
Human keratinocyte cell line HaCaT	LDH assay	24 h	>10 *μ*M for both Que and QAgNPs	[68]
Human macrophage cell line U937	Acid phosphatase assay	48 h	70 ± 10.7 *μ*M	[33]
Human monocyte cell line U937	Acid phosphatase assay	48 h	24.9 ± 3.5 *μ*M	[33]
Human promyelocytic leukemia cell line HL-60	Alamar Blue assay	72 h	51.3 ± 0.4 *μ*M	[27]
Human red blood cells	Measurement of Hb release	30 min	A hemolysis percentage of <1 at 1000 *μ*g/mL	[67]
Human red blood cells	Measurement of Hb release	3 h	A hemolysis percentage of <10 at 1000 *μ*g/mL	[43]
Monkey kidney cell line BGM	MTT assay	24 h	≥1000 *μ*g/mL	[55]
Madin-Darby canine kidney (MDCK) cell line	MTT assay	48 h	>100 *μ*M	[60]
Rat myoblast cell line L6	Alamar Blue assay	72 h	37.1 *μ*g/mL	[32]
Murine macrophage cell line J774.2	Trypan blue assay	72 h	125.44 *μ*M	[25]
Murine macrophage cell line J774	MTT assay	144 h	>1000 *μ*g/mL for both Que and QPNPs	[36]
Murine macrophage cell line RAW 264.7	Trypan blue assay	24 h	27.3 *μ*M	[41]
Murine peritoneal macrophages	MTT assay	24 h	No loss of viability at 48 and 70 *μ*M	[26]
Murine peritoneal macrophages	MTT assay	48 h	44.5 ± 1.7 *μ*M (13.3 ± 0.5 *μ*g/mL)	[19]
Murine peritoneal macrophages	Microscopic counting	48 h	1400 and 1600 *μ*M for Que and QAunp, respectively	[34]
Murine peritoneal macrophages	Alamar Blue assay	72 h	80.2 *μ*M	[20]
Hamster peritoneal macrophages	MTT assay	48 h	478 ± 89 *μ*M	[28]

CC_50_: the 50% cytotoxic concentration; Hb: hemoglobin; LDH: lactate dehydrogenase; QAgNPs: quercetin-conjugated silver nanoparticles; QAunp: quercetin-conjugated gold nanoparticles; QPNPs: quercetin-loaded poly-*ε*-caprolactone nanoparticles; Que: quercetin.

**Table 3:** List of studies concerned with in vivo efficacy of quercetin toward different protozoan pathogens. Animal models, routes of administration, methods, dosing regimens, and key findings of the relevant studies are also outlined.

Protozoan parasites	Identifiers	Animal models	Routes of Que administration	Methods/dosing regimens	Key findings	References
*Leishmania amazonensis*	MHOM/BR/75/Josefa	BALB/c mice	Oral (intragastric gavage)	Animals were subcutaneously infected in the ear pinna with GFP promastigotes. At 7 days p.i., animals were treated daily with Que (16 mg/kg) for 30 days	(i) Significant suppression of the parasite burden using Que compared with the untreated mice (*p* < 0.01) (ii) Reduction in lesion growth in mice receiving Que (iii) Superiority of Que over intraperitoneal SSG (8 mg/kg, twice a week) in reducing the parasite burden on day 68 of infection	[72]
	MHOM/77BR/LTB0016	BALB/c mice	Intralesional	Animals were subcutaneously infected in the footpad with promastigotes. At 15 days p.i., they received 5 doses of Que (30 mg/kg) every 4 days	(i) Increasing values of lesion size during the first 2 weeks of treatment in Que-treated animals (ii) Significant reduction in the parasite burden (*p* < 0.05) at 4 and 6 weeks p.i. in Que-treated mice as compared with the untreated group (iii) Superiority of Que over Glucantime® (30 mg/kg) in reducing the parasite burden at 4 and 6 weeks p.i. (iv) No signs of animal death and no evidence of body weight loss higher than 10% in the animals exposed to Que	[19]
	MHOM/BR/75/Josefa	BALB/c mice	Oral (intragastric gavage)	Animals were infected in the ear with GFP promastigotes and were given 51 daily oral doses of Que (16 mg/kg) or LNC-loaded Que (0.4 mg/kg)	(i) Reduction in the lesion sizes (38%) and parasite loads (71%) using Que (ii) Reduction in the lesion sizes (64%) and parasite loads (91%) using LNC-Que (iii) No evidence of treatment toxicity	[73]

*Leishmania braziliensis*	MCAN/BR/98/R619	Golden hamsters	Oral	Animals were infected in the dorsal hind paw with promastigotes and were treated with Que (20 mg/kg; five times a week) for 8 weeks starting on the 7th day of infection	(i) Reduction in the lesion thickness and parasite load in Que-treated hamsters as compared with the untreated group (ii) No evidence of treatment toxicity (iii) Lower in vivo efficacy of Que in comparison to 80 mg/kg of intraperitoneal Glucantime® (3 times a week, every other day)	[28]

*Leishmania donovani*	MHOM/IN/1983/AG83	Golden hamsters	Oral	Animals received Que (14 mg/kg) twice a week at 4 days p.i. with freshly purified amastigotes, and the treatment was continued for 4 weeks	(i) Reduction in the splenic parasite load by 90%	[29]
	MHOM/IN/1983/AG83	Golden hamsters	Oral	Que (5–40 mg/kg) was administered biweekly to 1-month-infected animals	(i) Suppressing the oxidation of lipids and proteins in the RBC membranes of infected animals in response to Que treatment (ii) Rectification of anemia during infection (increasing both Hb levels and RBC lifespan) in response to Que treatment (iii) Reduction in the spleen parasite load in Que-treated animals (iv) Superiority of SSG/Que combination for reduction of ^•^OH in RBCs, prevention of proteolytic degradation of bands 3 and 4.1 in RBC membranes, and decrements in osmotic fragility of RBCs compared to either agent alone	[74]
	MHOM/IN/1983/AG83	Golden hamsters	Oral	At 30 days p.i., animals received oral Que (20 mg/kg) thrice a week. Hamster Salb was also injected intravenously at the same dose twice a week. Treatment was continued for 4 weeks	(i) Increasing in vivo bioavailability of Que using Que/Salb combination (ii) Superiority of Que/Salb combination over Que in preventing the accumulation of heme iron, reducing ^•^OH in RBCs, and increasing both Hb levels and RBC lifespan	[75]
	MHOM/IN/1983/AG83	Golden hamsters	Oral	In the monotherapy study, animals received oral Que (5 to 50 mg/kg) thrice a week. In the combination therapy, Que was given orally and Salb was applied intravenously thrice a week (both at a dose of 20 mg/kg)	(i) Dose-dependent reduction in the splenic parasite burden using Que (ii) Increasing bioavailability of Que in animals subjected to the combined treatment (iii) Superiority of Que/Salb combination over Que in reducing the splenic parasite load (iv) Reduction in iron incorporation in the amastigotes collected from animals receiving the combined treatment (v) Disintegration of the amastigotes within phagolysosomes from the spleen in response to the combined treatment (vi) Remarkable reduction in the activity of ribonucleotide reductase in the amastigotes isolated from the Que/Salb-treated animals	[76]
	MHOM/IN/1983/AG83	Golden hamsters	Subcutaneous	Animals were intracardially infected with amastigotes. At 30 days p.i., they received either free Que or Que in vesicular forms (each contains 300 *μ*g of Que intercalated in 0.5 mL of different vesicular suspensions)	(i) EC_50_ value of 3 mg/kg body weight for Que (ii) Reductions in the spleen parasite burden at the above-mentioned dose: free Que (26%), Que-intercalated liposomes (51%), Que-intercalated niosomes (68%), Que-intercalated nanoparticles (87%), and Que-intercalated microspheres (44%) (iii) Increasing SGPT, AP, serum urea, and creatinine levels following Que treatment. All of these levels remained close to normal in response to different vesicular forms (iv) Reducing both hepatotoxicity and renal toxicity, especially using Que-intercalated nanoparticles	[77]
	MHOM/ET/67/L82	BALB/c mice	Intraperitoneal	Animals were infected with amastigotes (in a 0.2 mL bolus via a lateral tail vein). At 7 days p.i., they received Que (30 mg/kg) for 5 consecutive days	(i) Lower in vivo efficacy of Que in comparison to 30 mg/kg of oral miltefosine (15.3% vs. 96.6%; reduction in the hepatic parasite load)	[32]

*Leishmania major*	MHOM/DZ/2000/LIPA1126	BALB/c mice	Subcutaneous	Dorsal air pouches were raised on mice and were inoculated with promastigotes. The infected animals received Que (25 mg/kg) once daily for 4 days	(i) Reduction in inflammatory cell infiltration at 24 and 96 h p.i. (ii) Increasing the number of apoptotic neutrophils harboring apoptotic amastigotes at 24 and 96 h p.i. (iii) Restoring iNOS expression and activity via TNF-*α* stimulation in subcutaneous tissue at 24 and 96 h p.i.	[10]
	NM	BALB/c mice	Oral, intradermal, and intraperitoneal	Mice were intradermally infected with amastigotes. After appearance of wounds, the animal group received Que (14 mg/kg) twice a week for 4 weeks	(i) Decreasing the percentage of mortality in mice receiving Que through oral (40%), intradermal (60%), and intraperitoneal (57.14%) routes as compared to the placebo (100%) (ii) Regarding the recovery of mice from cutaneous leishmaniasis, no significant difference between Que-treated groups and Glucantime®-treated groups was observed (*p* = 1.00)	[78]
	MHOM/IR/75/ER	Wistar rats	Intraperitoneal	Animals with infected tails received Que (50 *μ*g/mL per kg) twice daily for 30 days	(i) Decreasing both lesion size and the number of amastigotes in mice on the 30th day of exposure to Que	[40]
	MRHO/IR/75/ER	BALB/c mice	Oral	Mice were injected subcutaneously at the tail base with *L. major*. Five weeks after inoculation, the infected mice received Que (50 mg/kg) once daily for 28 consecutive days	(i) Significant reduction in the lesion area in the Que-treated group as compared to the untreated group (*p* < 0.05) on the 12th day after the onset of treatment (ii) Reduction in the parasite load in the margin of cutaneous lesions in the Que-treated group (iii) Significant reduction in the number of inflammatory cells in the Que-treated group in comparison to the untreated group (*p* < 0.05) (iv) Formation of granulation tissue in the depth of ulcers in the Que-treated group (v) Significant increases in neovascularization, the number of both fibroblasts and fibrocytes, the levels of both FRAP and adiponectin, and GPX activity in the Que-treated group in comparison to the untreated group (*p* < 0.05) (vi) Significant reduction in the levels of MDA, TNF-*α*, and IL-6 in the Que-treated group in comparison to the untreated group (*p* < 0.05)	[41]
	MRHO/IR/75/ER	BALB/c mice	Topical	Mice were injected subcutaneously at the tail base with promastigotes and then received luteolin/AgNPs/Que ointment containing Vaseline, AgNPs/Que (0.5%), and luteolin (0.15%) every day for 21 days	(i) Increasing the lesion size in all cases (a slight increase in the group receiving the ointment, but a significant increase in the untreated group) (ii) Acceleration of wound healing by reducing the parasitic load and inflammatory responses, particularly in mice receiving the ointment (iii) Faster wound healing process in the ointment-treated group than the Glucantime®-treated group (300 mg/mL; injection)	[42]

*Plasmodium berghei*	NK65	Swiss mice	Oral	Mice were inoculated intraperitoneally with parasitized RBCs. After 24 h, they were treated by Que (50 mg/kg), with one daily dose for three consecutive days	(i) Reducing the parasitemia by 52% and 44% on days 5 and 7 in response to Que, respectively (ii) Lower in vivo efficacy of Que in reducing the parasitemia in comparison to CHQ (20 mg/kg)	[55]
	NM	BALB/c mice	Intraperitoneal	Mice were inoculated intraperitoneally with parasitized RBCs. These mice were treated with NQ (10 mg/kg body), alone or in combination with HF (2 mg/kg) for 4 days	(i) Significant reduction in histopathological damage in NQ-treated infected mice as compared to the nontreated infected mice (*p* < 0.05) (ii) Significant reduction in the levels of proinflammatory cytokines (IL-1*β* and TNF-*α*) in NQ-treated infected mice as compared to the nontreated infected mice (*p* < 0.05) (iii) Superiority of NQ/HF combination over NQ in reducing both histopathological damage of *P. berghei* and serum levels of proinflammatory cytokines	[79]
	NK65 and ANKA strains	ICR mice	Intraperitoneal	Mice were inoculated intraperitoneally with parasitized RBCs. These mice were treated with Que (2.5, 5, 10, 15, 25, and 50 mg/kg) for four consecutive days, starting at 1 h after the parasite inoculation on day 0	(i) Suppression of parasitemia development on day 4 and prolongation of median survival in NK65-infected mice (receiving 2.5 to 50 mg/kg Que) and ANKA-infected mice (receiving 15 to 50 mg/kg Que) (ii) Lower in vivo efficacy of Que in suppressing parasitemia development on day 4 in comparison to 10 mg/kg of CHQ (iii) Significant increase in GSK3*β* (Ser-9) phosphorylation in the liver of NK65-infected mice (*p* < 0.05 vs. control) (iv) Significant increase in GSK3*β* (Ser-9) phosphorylation in the brain of ANKA-infected mice (*p* < 0.05 vs. control) (v) Reducing proinflammatory cytokines TNF-*α* and IFN-*γ* in serums of NK65-infected mice on day 4 p.i. (vi) Increasing anti-inflammatory cytokines IL-10 and IL-4 in serums of NK65-infected mice on day 4 p.i.	[56]

*Plasmodium juxtanucleare*	Versiani and Gomes, 1941	White Leghorn chicken (*Gallus gallus* Linnaeus, 1758)	Oral gavage	Chickens were infected with *P. juxtanucleare* and then were immunocompromised by the administration of MP (26 mg/kg) in the pectoral muscle. These animals were treated with Que (50 mg/kg) for four consecutive days	(i) Significant reduction in parasitemia in Que-treated group in comparison to the control group (*p* < 0.01) (ii) Lower in vivo efficacy of Que in reducing parasitemia in comparison to oral CHQ (50 mg/kg)	[80]

*Toxoplasma gondii*	Two virulent (RH, ENT) and two avirulent (ME49, C) strains	BALB/c mice	Intraperitoneal and subcutaneous	Tachyzoites were treated with AntiA and Que (50 *μ*M) before injection into the peritoneal cavities of mice. Hsp70 expression was then assessed in *T. gondii* recovered from the peritoneal cavities. Moreover, spleens from subcutaneously infected mice (either treated or untreated) were collected to assess the parasite burden at 4 days p.i.	(i) Reduction in Hsp70 expression in tachyzoites of RH (87%), ENT (78%), ME49 (50%), and C strains (50%) recovering from the peritoneal cavities (ii) Reduction in the splenic burden of virulent strains (RH: 45%, ENT: 25%) with reduced Hsp70 expression (the treated virulent strains) as compared with the untreated virulent strains (iii) No significant difference in the splenic parasite burden between the untreated avirulent strains and those with reduced Hsp70 expression (the treated avirulent strains)	[59]

*Babesia microti*	Munich strain	BALB/c mice	Intraperitoneal	Mice were injected intraperitoneally with parasitized RBCs. These mice were treated with Que (14.5 mg/kg), and the parasitemia was then checked every day until day 22	(i) Significant reduction in parasitemia in the Que-treated group from days 4 to 8 p.i. in comparison to the control group (*p* < 0.01) (ii) Lower in vivo efficacy of Que in reducing parasitemia in comparison to diminazene aceturate (25 mg/kg; intraperitoneal)	[64]

AntiA: antisense oligonucleotides targeting the *T. gondii* Hsp70 ATG start codon; AP: alkaline phosphatase; CHQ: chloroquine; EC_50_: the drug concentration at which the parasite load of the spleen reduced to 50%; FRAP: ferric-reducing ability of plasma; GFP: green fluorescent protein; GPX: glutathione peroxidase; GSK3*β*: glycogen synthase kinase-3*β*; Hb: hemoglobin; HF: hydroxychloroquine sulfate; Hsp70: heat shock protein 70; IFN-*γ*: interferon-gamma; IL-6: interleukin 6, iNOS: inducible nitric oxide synthase; LNC: lipid-core nanocapsules made of a poly-*ε*-caprolactone shell; MDA: malondialdehyde; mg/kg: milligrams per kilogram of body weight; MP: methylprednisolone; NM: not mentioned; NQ: nanophytosomes of quercetin; ^•^OH: hydroxyl radical; p.i.: postinfection; Que: quercetin; RBC: red blood cell; Salb: serum albumin; SGPT: serum glutamate pyruvate transaminase; SSG: sodium stibogluconate (Pentostam®); TNF-*α*: tumor necrosis factor-alpha.

## Data Availability

The datasets generated during and/or analyzed during the current study are available from the corresponding author on reasonable request.
